# Combining Triazole Scaffold Repurposing and Generative Transformer Architecture for Structure-Based Inhibitor Design Targeting the LasR Quorum Sensing Receptor of *Pseudomonas aeruginosa*

**DOI:** 10.3390/ph19081269

**Published:** 2026-08-11

**Authors:** Abbas Khan, Muhammad Ammar Zahid, Anwar Mohammad, Asia Al-Jabiry, Raed M. Al-Zoubi, Mohanad Shkoor, Ameera Al-Jabiry, Abdelali Agouni

**Affiliations:** 1 Department of Pharmaceutical Sciences, College of Pharmacy, QU Health, Qatar University, Doha P.O. Box 2713, Qatar; 2 Precision Health Analysis Unit, Translational Research, Dasman Diabetes Institute, Dasman, Kuwait City P.O. Box 11803, Kuwait; anwar.mohammad@dasmaninstitute.org; 3 Department of Chemistry and Earth Sciences, College of Arts and Sciences, Qatar University, Doha P.O. Box 2713, Qatarmshkoor@qu.edu.qa (M.S.); 4 Surgical Research Section, Department of Surgery, Hamad Medical Corporation, Doha P.O. Box 3050, Qatar; 5 Department of Biomedical Sciences, College of Health Sciences, QU Health, Qatar University, Doha P.O. Box 2713, Qatar; 6 Department of Chemistry, Jordan University of Science and Technology, P.O. Box 3030, Irbid 22110, Jordan

**Keywords:** *Pseudomonas aeruginosa*, quorum sensing inhibition (LasR target), AI-driven drug discovery, structure-based virtual screening, anti-virulence therapeutics

## Abstract

**Background:** The rapid escalation of multidrug-resistant *P. aeruginosa* necessitates anti-virulence strategies targeting quorum sensing rather than bacterial survival; however, integrating scaffold repurposing with generative AI to inhibit LasR remains underexplored. Here, we address this gap by combining triazole scaffold mining with transformer-based de novo molecular generation to systematically identify putative LasR inhibitors. **Methods:** An integrated computational pipeline involving Structure-based inhibitor design using Generative Transformer Architecture, deep learning-assisted GNINA rescoring, density functional theory optimization, and molecular dynamics simulations was employed, followed by MM-GBSA binding free energy estimation. **Results:** Screening of 2666 triazole derivatives and 19,861 DrugGPT-generated compounds yielded top hits with superior binding affinities (−11.59 to −13.81 kcal/mol) compared to the reference ligand (−8.50 kcal/mol). MD simulations yielded stable protein–ligand complexes with RMSD values of 2.24–3.01 Å, while key interactions involving residues Tyr50, Asp67, and Ser123 were consistently maintained. Binding free energy calculations further confirmed strong thermodynamic stability, with MM-GBSA ΔG_bind_ values significantly favorable, supporting robust ligand–receptor affinity. **Conclusions:** Collectively, these findings establish a powerful AI-integrated framework for anti-virulence drug discovery and identify structurally diverse, high-affinity triazole-based and de novo compounds as promising lead candidates for disrupting LasR-mediated quorum sensing in *P. aeruginosa*.

## 1. Introduction

*Pseudomonas aeruginosa* (*P. aeruginosa*) is a Gram-negative opportunistic pathogen that is a significant cause of hospital- and healthcare-associated infections worldwide [1]. This bacterium can be found in many environmental sources, including soil, water, and hospital equipment, leading to frequent transmission between hospitals and patients [2]. *P. aeruginosa* is associated with many types of infections in a clinical setting, including ventilator-associated pneumonia (VAP), urinary tract infections (UTIs), infections of burns and wounds, bacteremia and septicemia, as well as being a significant opportunistic pathogen among patients who are immunocompromised or have chronic diseases, such as cystic fibrosis (CF) [1]. In CF patients, the progression of lung disease and increased mortality occur due to chronic colonization of the respiratory tract with *P. aeruginosa* [3]. The ability of *P. aeruginosa* to resist a variety of antibiotics through both intrinsic mechanisms, such as multidrug efflux pumps, and acquired mechanisms, including enzyme degradation, reduced outer membrane permeability, and biofilm-associated adaptive resistance, underscores its clinical importance [4]. Biofilms form when these bacteria grow together in a polysaccharide layer within the extracellular polymeric substance, providing protection from host immunity and blocking the effectiveness of antibiotics, thereby allowing *P. aeruginosa* to persist in chronic and recurrent infections [5]. Due to these characteristics, *P. aeruginosa* has been designated by the World Health Organization as a “critical priority pathogen,” requiring the urgent development of new antibiotics and therapeutic strategies [6]. The rise in the prevalence of multidrug-resistant (MDR) and extensively drug-resistant (XDR) strains has made the treatment of infections difficult, ultimately increasing morbidity and mortality rates [7]. As a result, new therapeutic options target bacterial virulence mechanisms rather than viability, representing a new strategy for treating primary infections caused by *P. aeruginosa*.

One of the essential virulence mechanisms in *P. aeruginosa* is the quorum-sensing (QS) system, which enables cell-to-cell communication based on cell density. This cell-density-dependent communication uses small, diffusible molecules called autoinducers to regulate the expression of virulence factors once a threshold population density is reached. *P. aeruginosa* has multiple interconnected quorum systems, with the Las quorum system (master regulator) controlled by the transcriptional regulator LasR and its autoinducer, N-3-oxododecanoyl-L-homoserine lactone (3-oxo-C12-HSL), being the master regulator of virulence gene regulation. When it binds its signaling molecule, LasR activates the expression of many genes encoding virulence factors, including elastase, exotoxin A, proteases, pyocyanin, and factors involved in biofilm formation and bacterial motility [8]. Together, these virulence factors result in tissue damage, avoidance of immune responses, and continued establishment of chronic infections. Disruption of LasR signaling has been shown in experimental studies to significantly diminish the pathogenicity of *P. aeruginosa* in various animal models of infection, and inhibition of this regulatory pathway can decrease bacterial virulence without concomitantly decreasing bacterial growth [9]. Therefore, LasR is being extensively studied for potential use as an anti-virulence drug target. Many small molecules have been identified that inhibit LasR activity, including analogs of acyl-homoserine lactones (AHLs), halogenated furanones, and plant-derived flavonoids, which can prevent either ligand binding to LasR or transcriptional activation of LasR-target genes [10]. For instance, a brominated furanone purified from marine algae has been shown to inhibit quorum sensing and biofilm production by *P. aeruginosa*, underscoring the potential of quorum-sensing inhibitors as therapeutic agents [11]. These data support the development of novel compounds that block LasR signaling to reduce virulence and prevent the emergence of antibiotic-resistant strains.

Among the diverse chemical scaffolds explored in antimicrobial drug discovery, Triazole derivatives have become popular scaffolds for the development of new antimicrobial agents due to their diverse biological activities and good pharmacological properties [12]. The triazole ring, a five-membered aromatic heterocycle containing three nitrogen atoms, can form a range of different associations with biological targets through hydrogen bonding, π–π stacking, and metal coordination [13]. Due to this range of attachment sites, triazoles are valuable scaffolds for the design of new drugs in medicinal chemistry. Triazole-containing drugs have been extensively used as antifungal agents, including commercially available compounds such as fluconazole, itraconazole, and voriconazole, which inhibit fungal cytochrome P450 enzymes involved in ergosterol synthesis [14]. In addition to their antifungal use, many triazole derivatives have also been shown to exhibit significant antibacterial activity against various Gram-positive and Gram-negative bacterial species, including *Staphylococcus aureus*, *Escherichia coli*, and *P. aeruginosa* [15]. Triazole-based molecules synthesized via click chemistry demonstrate considerable promise as inhibitors of bacterial enzymes and virulence gene regulators, owing to their structural diversity and ease of synthesis [16]. Several studies have described the ability of certain triazole derivatives to disrupt bacterial biofilm formation and inhibit bacterial quorum-sensing pathways, suggesting that triazole-derived compounds could be useful agents for the development of new anti-virulence therapies [17]. In particular, triazole-based molecules synthesized via click chemistry have shown promising inhibitory effects against bacterial enzymes and virulence regulators, owing to their structural diversity and synthetic accessibility. Several studies have reported triazole derivatives that can disrupt bacterial biofilms and inhibit quorum-sensing pathways, highlighting their potential as anti-virulence agents. The ability of triazole scaffolds to be easily functionalized further allows rational optimization of molecular interactions with protein targets. With the increasing availability of structural information on bacterial virulence regulators such as LasR, triazole derivatives represent a promising class of compounds for structure-based drug discovery targeting quorum-sensing inhibition [18]. However, identifying potent and selective inhibitors requires efficient screening strategies that can explore large chemical spaces and optimize ligand–target interactions.

In recent years, computer-aided drug design (CADD) and artificial intelligence (AI) in drug discovery (AIDD) have dramatically advanced pharmaceutical research, enabling rapid identification and optimization of bioactive molecules. CADD uses computational methodologies such as molecular docking, virtual screening, pharmacophore modeling, and molecular dynamics simulations to predict how ligands will interact with their targets and to evaluate the stability of compounds before conducting any laboratory work [19,20,21]. These methodologies allow us to greatly reduce both the time and cost associated with traditional drug discovery pipelines while simultaneously improving the efficiency of identifying potential drug candidates. In addition, through the use of AI and machine learning, our capabilities in computational drug discovery have increased through predictive modeling and the generation of new molecules *de novo*. For example, deep learning architectures, such as transformer-based generative models, can analyze large chemical datasets to identify structure–activity relationships and generate new molecules optimized for specific targets [22]. These methodologies have already produced numerous success stories in the discovery of new antimicrobial drugs, including the identification of novel compounds that exhibit efficacy against antibiotic-resistant organisms through machine-learning-guided screening [23].

**Figure 1:**
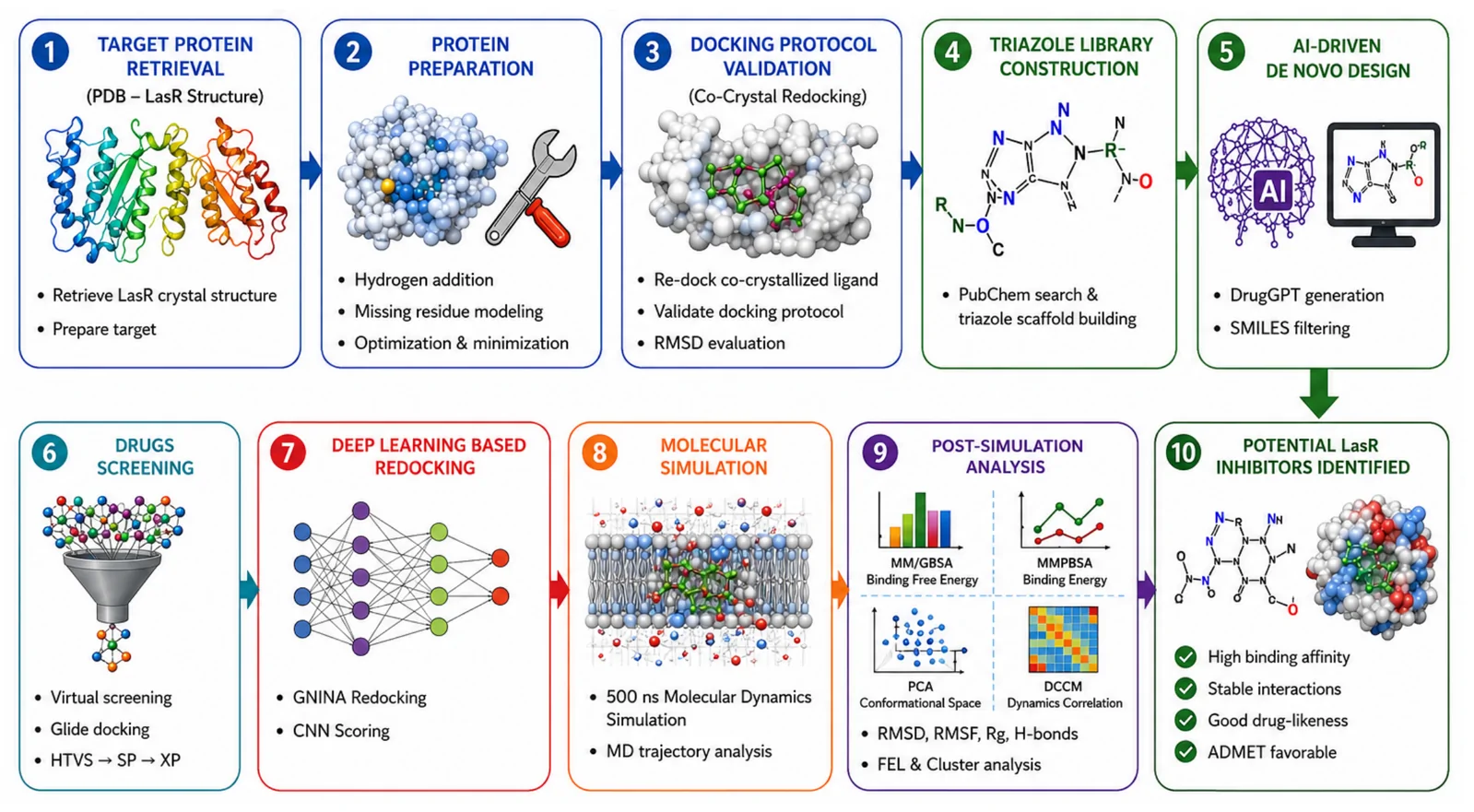
Systematic workflow for the whole work. The workflow contains multiple steps from protein retrieval to final validation.

In the present study, an integrated computational strategy combining substructure-based compound retrieval, transformer-based generative molecular design, structure-based virtual screening, and molecular dynamics simulations was employed to identify potential inhibitors of the LasR quorum-sensing receptor in *P. aeruginosa*. The PubChem database was queried for triazole-based compounds due to their known antimicrobial activity, and a transformer-based generative model (DrugGPT) was then used to design target-aware molecules, providing a new source of chemical space for investigation. The triazole-based and the newly designed compounds were then screened through virtual docking and validated via molecular simulation against the LasR binding domain. These putative novel molecules could disrupt LasR-mediated quorum sensing and thereby contribute to the development of alternative therapeutic strategies targeting virulence pathways in multidrug-resistant *P. aeruginosa*. The whole workflow of the work is shown in Figure 1.

## 2. Results and Discussion

### 2.1. Virtual Screening of Triazole Derivatives Against LasR

A hierarchical Schrödinger Virtual Screening Workflow (VSW) was employed to identify promising triazole-based LasR binders from the screened library. The initial library contained 2666 input structures, which were reduced to 266 compounds after HTVS, then to 26 compounds after SP docking, and finally to 4 top-ranked structures after XP docking, all of which proceeded to MM-GBSA evaluation. The high-scoring compounds were systematically excluded, and the high-affinity candidates with the lowest docking scores were retained after XP docking. Among these, based on the XP GScores for best hits ranging from −11.33 to −13.81 kcal/mol were reported. The top-ranked hits include CID124147019 with the docking score of −11.70 kcal/mol, CID141801554 with the docking score of −11.59 kcal/mol, and the final hit CID67886800 with −11.33 kcal/mol. Notably, all shortlisted compounds outperformed the reference control (−8.50 kcal/mol) by a substantial margin, showing higher predicted binding affinity. Thus, these results indicate that the screened triazole scaffolds are highly compatible with the LasR binding pocket and could therefore produce stronger inhibitory effects in the experiments than the control. Using the stringent XP Scoring Protocol criteria, this small subset of candidate compounds exhibits higher binding affinity than the control ligand and therefore could be novel putative computational hits, pending experimental proof of biological activity and binding affinity.

### 2.2. Virtual Screening of DrugGPT-Designed Compounds Against LasR

To further investigate the AI-assisted *de novo* designed inhibitor library, the DrugGPT-generated molecular library was subjected to a multistage virtual screening workflow targeting the LasR binding pocket. Initially, DrugGPT generated 241,450 molecular structures across 4924 generation batches, from which 19,861 chemically valid compounds were retained following structural sanitization, duplicate removal, and physicochemical filtering. Subsequent Lipinski and drug-likeness filtering reduced the library to 13,359 compounds suitable for hierarchical virtual screening. The Glide-based screening protocol progressively enriched the dataset, retaining 956 compounds after HTVS, 10 after SP docking, and 6 high-confidence candidates after XP docking and MM-GBSA refinement. The final shortlisted compounds demonstrated substantially stronger predicted binding affinities than the reference ligand (XP docking score: −8.50 kcal/mol). Among the top-performing DrugGPT-derived molecules, DrugGPT M1 exhibited the strongest XP docking score of −13.81 kcal/mol with a GNINA score of −10.479, followed by DrugGPT M2 with an XP score of −12.42 kcal/mol and GNINA score of −9.902, while DrugGPT M3 retained favorable binding with an XP docking score of −12.11 kcal/mol and GNINA score of −9.721 kcal/mol. The consistent enrichment observed throughout the hierarchical screening workflow suggests that the AI-guided molecular generation process successfully captured key structural and physicochemical determinants associated with LasR binding. These computationally prioritized hit candidates were subsequently advanced to molecular dynamics simulations and post-simulation conformational stability assessment to further evaluate their inhibitory potential. The best hits identified from both the Triazoles, and *de novo* designed groups are provided in Table 1.

### 2.3. Interaction Analysis of the Reference Ligand

**Figure 2:**
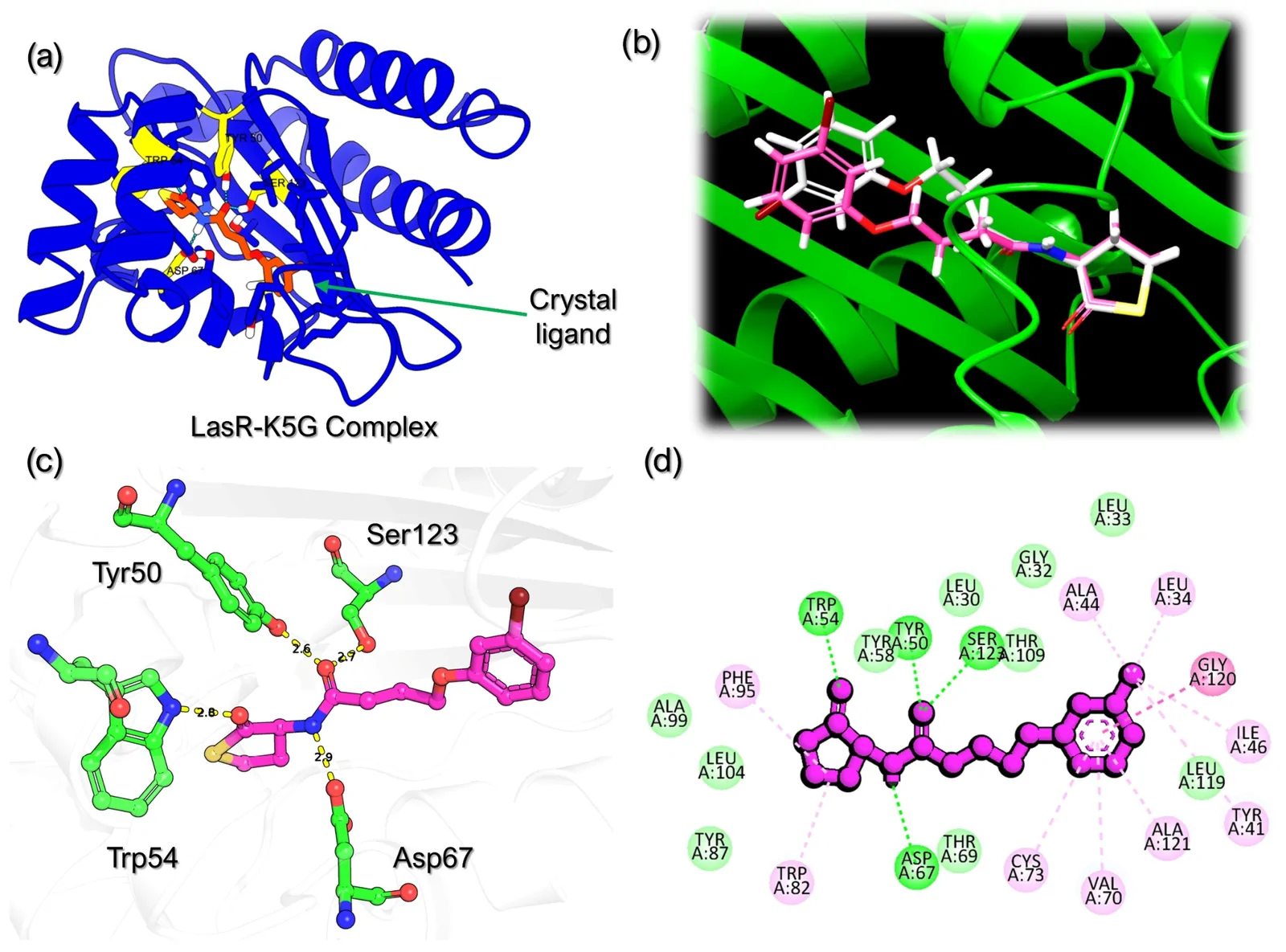
The co-crystallized structure of the LasR from Pseudomonas aeruginosa, along with the interaction pattern of the reference ligand. (**a**) shows the crystallographic coordinates of LasR, retrieved from RCSB, with PDB ID 6MWL. The orange stick represents the crystal ligand (K5G) along with the key interacting residues in yellow sticks. (**b**) shows the redocked control ligand superimposed on the co-crystal and (**c**) shows the 3D interaction patterns, while (**d**) shows the 2D interaction pattern by establishing hydrogen bonds shown in green, hydrophobic, and pi-pi interactions with the other key residues.

The interaction profile of the co-crystallized ligand within the LasR binding pocket reveals a well-defined and functionally relevant binding mode (Figure 2a). The co-crystallized LasR is deeply embedded in the receptor’s hydrophobic core, stabilized by a combination of hydrogen bonding, π-π interactions, and extensive hydrophobic contacts. The reference ligand has established multiple key hydrogen-bonding interactions with Asp67, Tyr50, Trp54, and Ser123, which are critical residues involved in ligand recognition and stabilization at the LasR active site. Specifically, Asp67 binding appears to be anchored within the conserved area of the binding pocket. Similarly, Tyr50 and Ser123 provide directional stabilization while Trp54 stabilizes the other tail of the ligand scaffold in the active site. The majority of the binding interaction comprises hydrophobic interactions involving the following hydrophobic (non-polar) residues: Trp54, Leu30, Leu36, Ile46, Val70, and Phe95 (Figure 2b,c). These hydrophobic residues form a compact nonpolar “pocket” surrounding the ligand backbone. Furthermore, the presence of Trp54 enables π–π stacking interactions, thereby increasing ligand affinity and structural complementarity. Additionally, the 2D interaction map (Figure 2d) indicates that all co-crystallized ligands have established a balance of interactions, with polar interactions providing binding specificity and hydrophobic interactions providing binding stability. Finally, the co-crystallized ligand is centrally located and binds in a way that strongly corroborates with prior reports on the ligand-binding architecture of the LasR receptor.

**Figure 3:**
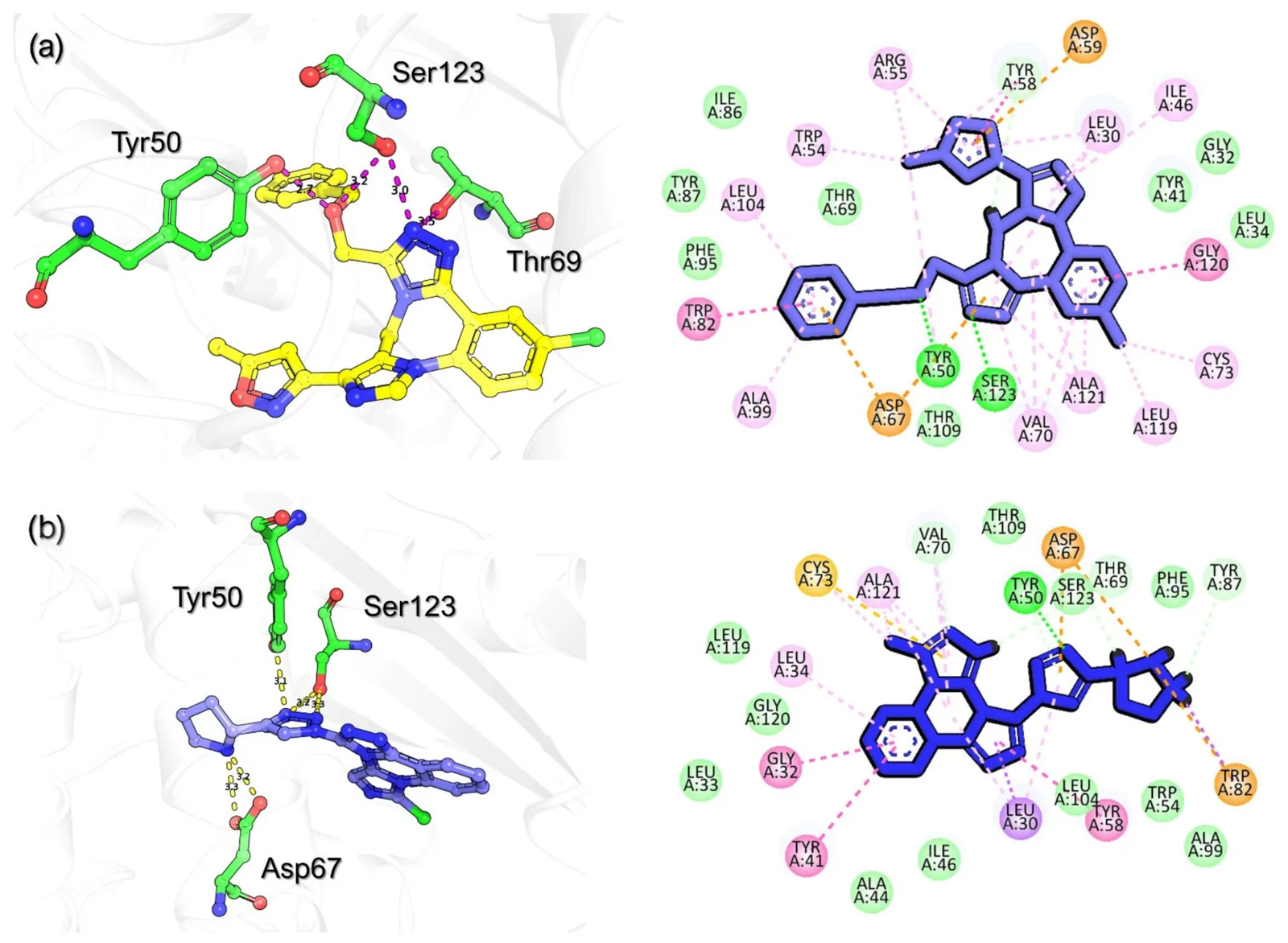
Interaction profiles of Triazoles (1 and 2) are shown in 3D and 2D maps. (**a**) shows the 3D and 2D interaction profiles of the Triazole Derivative 1, highlighting key hydrogen bonds and hydrophobic and aromatic interactions, such as pi-pi, in different colors. (**b**) shows the 3D and 2D interaction profiles of the Triazole Derivative 2, highlighting key hydrogen bonds and hydrophobic and aromatic interactions, such as pi-pi, in different colors.

To understand the binding pattern of Triazole Derivative 1 (TD1), the interactions were drawn in 3D and 2D, as shown in Figure 3a. The TD1 is well-positioned within the binding pocket, stabilized by several hydrogen bonds involving Tyr50, Thr69, and Ser123, with bond distances of ~2.0–3.5 Å. On the other hand, the 2D interaction map also confirms hydrogen bonding to Tyr50 and Ser123. Among the other types of interactions, including the hydrophobic contacts involving Leu30, Val70, Ala99, and Ile46, suggests that the ligand is stabilized through contacts with nonpolar residues. Moreover, the aromatic interactions are primarily established by Asp59 and Asp67, which are also the stabilizing factors by establishing pi-pi contacts with the ligand. The ligand appears well-oriented within the pocket, with its polar groups directed toward hydrogen-bonding residues and its aromatic rings positioned within hydrophobic regions. Unlike the TD1, Triazole Derivative 2 (TD2) formed a hydrogen-bonding network in the 3D representation, with interactions primarily involving Tyr50, Asp67, and Ser123. The 2D diagram confirms hydrogen bonding with Tyr50, Asp67, and Ser123, while hydrophobic interactions are observed with Leu30, Leu34, Tyr41, and Tyr58. Aromatic interactions are indicated with Asp67 and Trp82, suggesting possible π–π or π–alkyl interactions. The 3D and 2D interaction profiles for the TD2 are shown in Figure 3b. The ligand appears partially exposed, with fewer interactions compared to Triazole Derivative 1. The interaction pattern suggests stabilization through a combination of limited hydrogen bonding and hydrophobic contacts.

**Figure 4:**
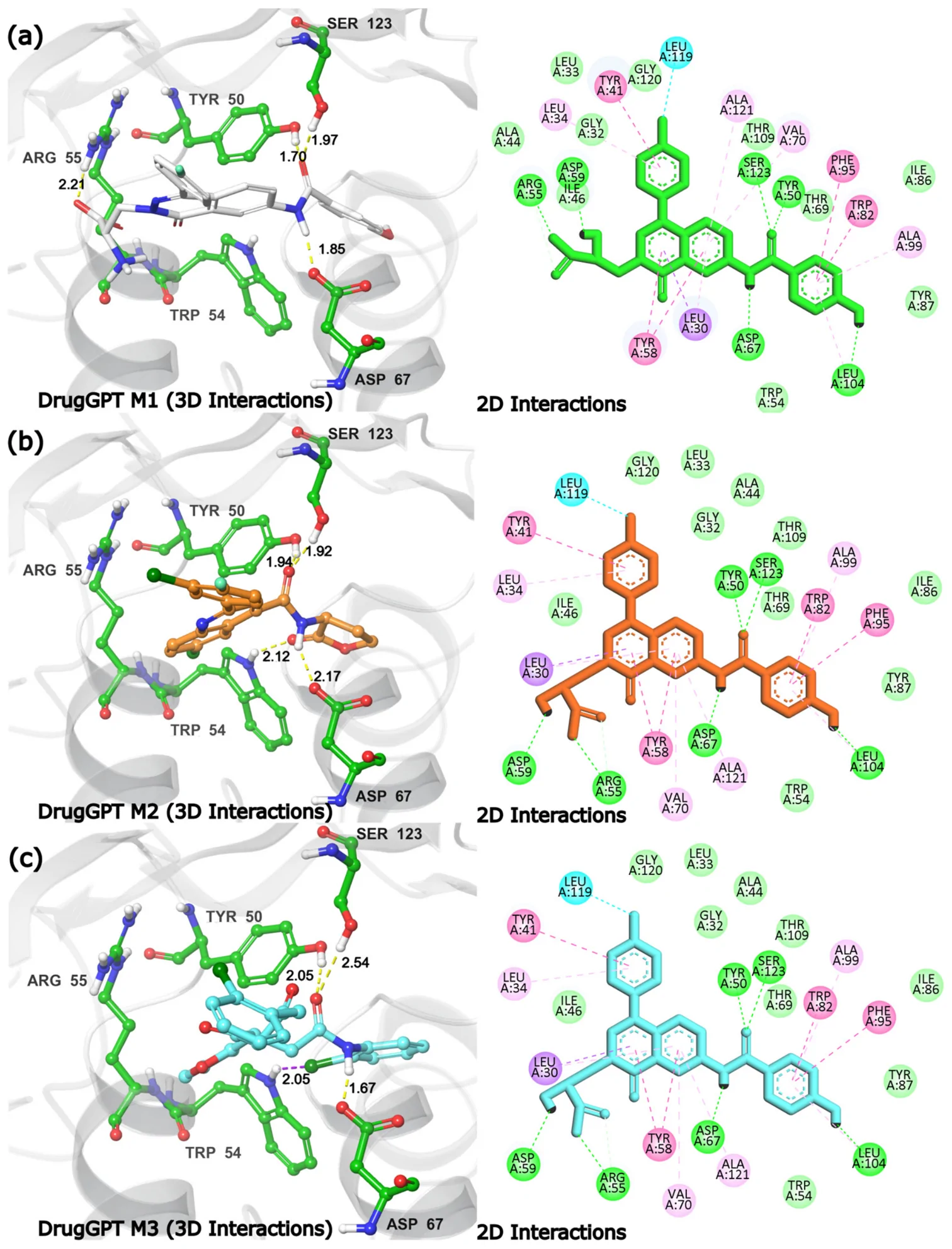
Interaction profiles of DrugGPT-designed molecules (1–3) shown in 3D and 2D maps. (**a**) shows the 3D as well as the 2D interaction profiles of the DG-1 with the key hydrogen bonds and hydrophobic and aromatic interactions, such as pi-pi, with different colors. (**b**) shows the 3D as well as the 2D interaction profiles of the DG-2 with the key hydrogen bonds and hydrophobic and aromatic interactions, such as pi-pi with different colors, while (**c**) shows the 3D as well as the 2D interaction profiles of the DG-3 with the key hydrogen bonds and hydrophobic and aromatic interactions, such as pi-pi with different colors.

To understand the binding of DrugGPT M1, it is positioned within a well-defined binding pocket, as shown in both 2D and 3D representations in Figure 4a. The interaction profile of DrugGPT M1 reported several hydrogen bonds involving Tyr50, Arg55, Asp67, and Ser123, shown in yellow dashed lines. Thus, this ligand DrugGPT M1 is anchored within the binding cavity through an enriched polar interaction network distributed across the binding region. The 2D interaction map, on the other hand, confirms hydrogen bonding with Tyr50, Arg55, Asp67, and Ser123, while additional hydrophobic, aromatics and π-type interactions were also reported, established by different residues including Leu30, Val70, Tyr58, Trp82, Phe95, Ala99, Leu104, and others shown in different dashed lines. In sum, these interactions stabilized the DrugGPT M1 within the LasR binding pocket. On the other hand, DrugGPT M2 occupies the same binding cavity and reports the same interaction pattern by establishing key hydrogen bonds with Tyr50, Arg55, Asp67, and Ser123 residues, which are essentially involved in the ligand interaction (Figure 4b). The 2D diagram showed additional hydrophobic and aromatic interactions involving Leu30, Val70, Tyr58, Trp82, Phe95, Ala99, and Leu104. The ligand configuration supports stable accommodation of the ligand within the LasR pocket. Similarly, DrugGPT M3 exhibits an interaction profile involving Tyr50, Arg55, Asp67, and Ser123 in hydrogen bonding with distances annotated (~1.67–2.54 Å), indicating typical hydrogen bond lengths and pattern as the previous ligands. The 2D diagram shows hydrogen bonding and additional interactions, including those with Asp59 and Leu104, as well as hydrophobic interactions involving Leu30, Leu34, Tyr41, Val70, Phe95, Trp82, and Leu104. The binding mechanism of DrugGPT M3 is shown in Figure 4c in both 3D and 2D.

In sum, residues including Tyr50, Arg55, Asp67, Trp82, and Ser123 were observed to be the most frequently interacting residues, and the *de novo*-designed ligands exhibit the strongest and most extensive bonding network, compared to the triazole derivatives and the control ligand enhanced by the presence of both polar and non-polar interactions, along with hydrogen bonds and aromatic/hydrophobic interactions. These top hits were then subjected to dynamic investigations to determine their binding stability and strength in a broader context.

### 2.4. Molecular Dynamics Simulation Analysis of Protein–Ligand Complexes

#### 2.4.1. Root Mean Square Deviation (RMSD)

**Figure 5:**
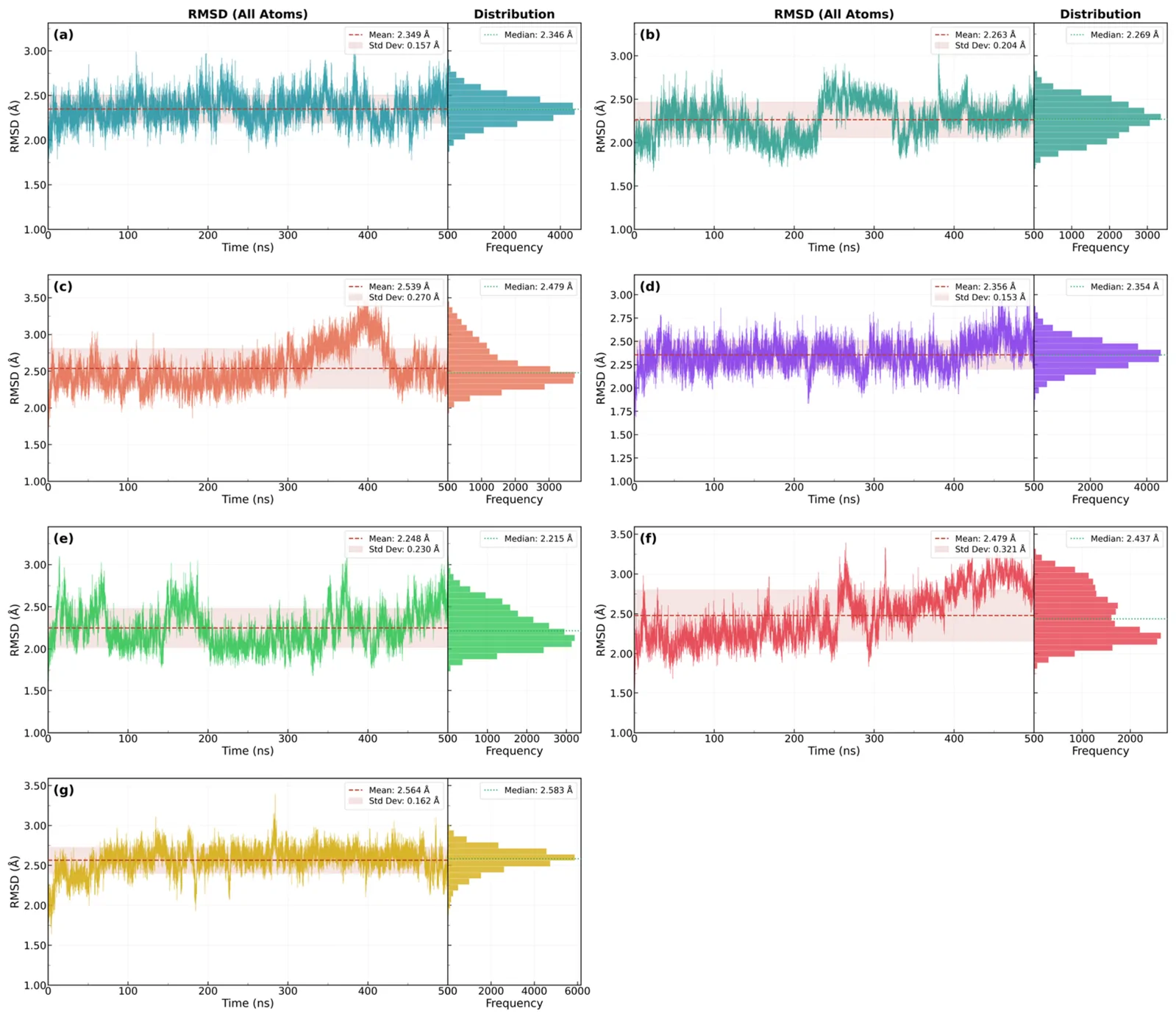
Structural stability assessment of the ligands bound to LasR in a dynamic environment. (**a**) shows the RMSD graph for the LasR-control complex with the mean RMSD 2.349 Å, (**b**) shows the RMSD graph for the LasR-DrugGPT M1 complex with the mean RMSD 3.023 Å, (**c**) shows the RMSD graph for the LasR-DrugGPT M2 complex with the mean RMSD 2.547 Å, (**d**) shows the RMSD graph for the LasR-DrugGPT M3 complex with the mean RMSD 2.573 Å, (**e**) shows the RMSD graph for the LasR-TD-1 complex with the mean RMSD 2.2.248 Å, (**f**) shows the RMSD graph for the LasR-TD-2 complex with the mean RMSD 2.479 Å while (**g**) shows the RMSD graph for apo system.

The structural stability of all six LasR–ligand complexes was assessed using backbone RMSD analysis across the 500 ns MD simulation trajectories, with the control complex (6MWL) as the reference. The control system shown in Figure 5a demonstrated good structural stability, converging rapidly to a mean RMSD of 2.349 ± 0.157 Å (median: 2.346 Å), with a narrow distribution indicative of a well-equilibrated, conformationally stable conformation after almost 20 ns. In the context of the LasR-DrugGPT M1 complex (Figure 5b), the RMSD profile remained comparatively stable throughout the simulation, despite conformational transitions observed between approximately 200 and 350 ns, and the system then subsequently re-equilibrated and maintained fluctuations largely within the 2.0–2.8 Å range, indicating moderate structural flexibility rather than major destabilization of the binding pocket. The mean RMSD for this complex, LasR-DrugGPT M1, was reported to be 2.263 ± 0.204 Å, with a median of 2.269 Å. Unlike the DrugGPT M1 complex, the LasR-DrugGPT M2 complex shown in Figure 5c displayed a mean RMSD of 2.539 ± 0.270 Å (median: 2.479 Å), with a noticeable gradual increase in RMSD after approximately 300 ns, followed by partial recovery after 400ns toward the end of the trajectory. On the other hand, the LasR-DrugGPT M3 complex showed one of the most stable and equilibrated RMSD profiles among all ligand-bound systems, with a mean RMSD of 2.356 ± 0.153 Å (median: 2.354 Å). The trajectory displayed highly uniform fluctuations throughout the simulation, with no substantial drift or transition states, suggesting that DrugGPT M3 maintains a compact, conformationally restrained binding mode that is highly comparable to the control complex. The RMSD graph for the LasR-DrugGPT M3 complex is shown in Figure 5d.

Unlike the previous complexes, the LasR-TD-1 complex shown in Figure 5e achieved the lowest mean RMSD of all six systems at 2.248 ± 0.230 Å (median: 2.215 Å), slightly below the control, but shows transient conformational changes implying moderate structural flexibility rather than significant destabilization of the binding pocket. The LasR-TD-2 complex displayed the average RMSD shown in Figure 5f and reported a mean RMSD of 2.479 ± 0.321 Å (median RMSD 2.437 Å), with greater temporal fluctuations than the control, indicating moderate instability during the last part of the simulation. A gradual rise in RMSD after about 250 ns was observed, as indicated by the frequency of large fluctuations and an expanded distribution, indicating that the conformational mobility of the complex was enhanced and structural restraint was reduced in relation to other ligand-bound systems. Finally, the apo LasR system, in Figure 5g, exhibited an average RMSD value of 2.564 ± 0.162 (median: 2.583 Å), a relatively stable trajectory after equilibration, while sampling somewhat larger RMSD values than the ligand-bound control system. This lack of stabilizing ligand interactions is expected to have helped explain the relatively greater conformational freedom of the apo protein observed throughout the simulation. In summary, whereas the control complex shows very tight conformation, certain ligand-bound systems exhibit increased RMSD and wider fluctuation profiles, showing ligand-induced conformational perturbations in the LasR binding pocket. Such controlled deviations from the native state suggest modulation of the receptor’s conformational dynamics rather than complete destabilization of the protein architecture and that the selected ligands exert their inhibitory effect by modulating LasR dynamic behavior and conformational flexibility relative to both the control and apo systems.

#### 2.4.2. Radius of Gyration (Rg)

**Figure 6:**
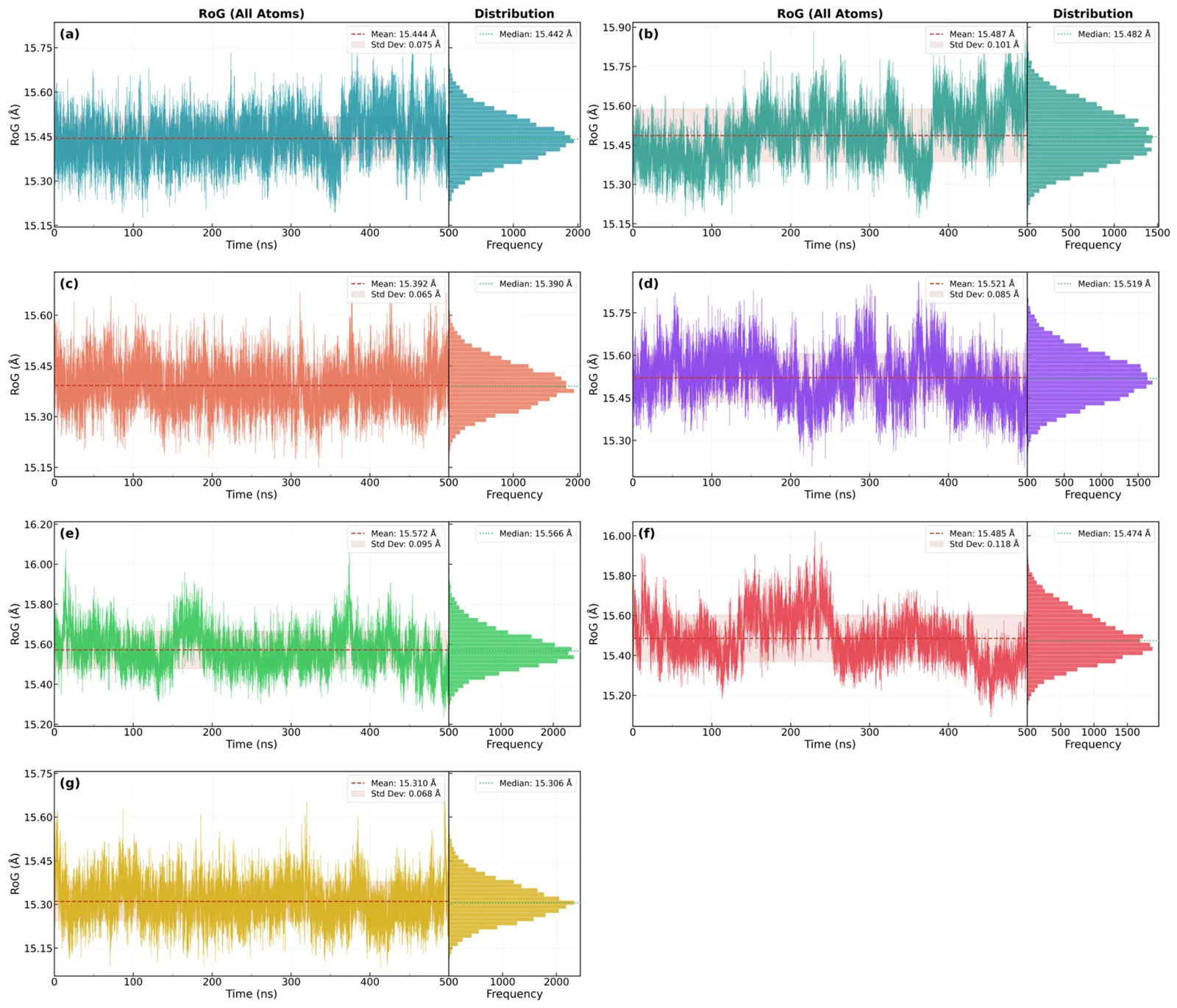
Structural folding analysis of the ligands bound to LasR in a dynamic environment. (**a**) shows the Rg graph for the LasR-control complex with the mean Rg 15.444 Å, (**b**) shows the Rg graph for the LasR-DrugGPT M1 complex with the mean Rg 15.863 Å, (**c**) shows the Rg graph for the LasR-DrugGPT M2 complex with the mean Rg 15.443 Å, (**d**) shows the Rg graph for the LasR-DrugGPT M3 complex with the mean Rg 15.450 Å, (**e**) shows the Rg graph for the LasR-TD-1 complex with the mean Rg 15.572 Å, (**f**) shows the Rg graph for the LasR-TD-2 complex with the mean Rg 15.485 Å, while (**g**) shows the Rg graph over the simulation time for the apo protein.

The global compactness and structural folding/unfolding patterns of each protein–ligand complex were assessed using radius of gyration (Rg) analysis throughout the entire 500 ns trajectory. As in the case of RMSD, a uniform, compact conformation of the LasR-control complex (Figure 6a) was observed with an average Rg of 15.444 Å, indicating that the protein maintained its folded architecture without any observable expansion or contraction throughout the simulation. Compared with the reference compound, compactness profiles of the LasR-DrugGPT M1 complex remained relatively stable over the simulation time, with moderate variations in the mean Rg value 15.487 ± 0.101 Å (median: 15.482 Å), but transient increases in Rg followed by rapid recovery toward equilibrium values while preserving the overall folded protein architecture. The Rg graph for the LasR-DrugGPT M1 is given in Figure 6b. A stronger alignment between LasR-DrugGPT M2 and control systems was observed throughout their respective simulations, with the mean Rg for LasR-DrugGPT M2 confirmed to be 15.392 ± 0.065 Å (median: 15.390 Å), and with a narrow distribution profile closely comparable to the control system, providing very strong evidence that LasR-DrugGPT M2 retains native protein compactness throughout the entire trajectory. The RG time series graph for LasR-DrugGPT M2 is shown in Figure 6c. On the other hand, the mean Rg of LasR-DrugGPT M3 was observed to be relatively higher than the control (15.521 ± 0.085 Å; median: 15.519 Å), but still compact and within the acceptable range. The RG time series graph for the LasR-DrugGPT M3 is shown in Figure 6d.

The LasR-TD-1 complex reported a relatively high average Rg of 15.572 ± 0.095 Å (median: 15.566 Å), consistently above the control throughout the simulation, suggesting a mild but continuous increase in the structural packing was observed. The Rg graph for the LasR-TD-1 complex is shown in Figure 6e. In contrast, the mean Rg for the LasR-TD-2 complex was reported to be 15.485 ± 0.118 Å (median, 15.474 Å). The trajectory showed larger variations and a more gradual reduction in Rg in the latter part of the simulation, particularly after about 250 ns, reflecting the late-stage structural compactness. The Rg graph for the LasR-TD-2 is given in Figure 6f. Finally, the apo LasR system shown in Figure 6g exhibited the lowest mean Rg value among all systems at 15.310 ± 0.068 Å (median: 15.306 Å), maintaining a highly compact and stable conformational profile throughout the simulation. The apo trajectory showed minimal fluctuations and a narrow distribution, indicating that the unbound protein remained structurally compact, free of ligand-induced conformational perturbations. In sum, compared with the apo protein, several ligand-bound complexes showed mildly increased Rg values, suggesting that ligand binding induces moderate conformational adaptation within the receptor while maintaining overall structural integrity. These observations are consistent with the RMSD analysis, which showed ligand-induced dynamic modulation rather than severe destabilization of the receptor structure relative to both the control and apo systems.

#### 2.4.3. Root Mean Square Fluctuation (RMSF)

**Figure 7:**
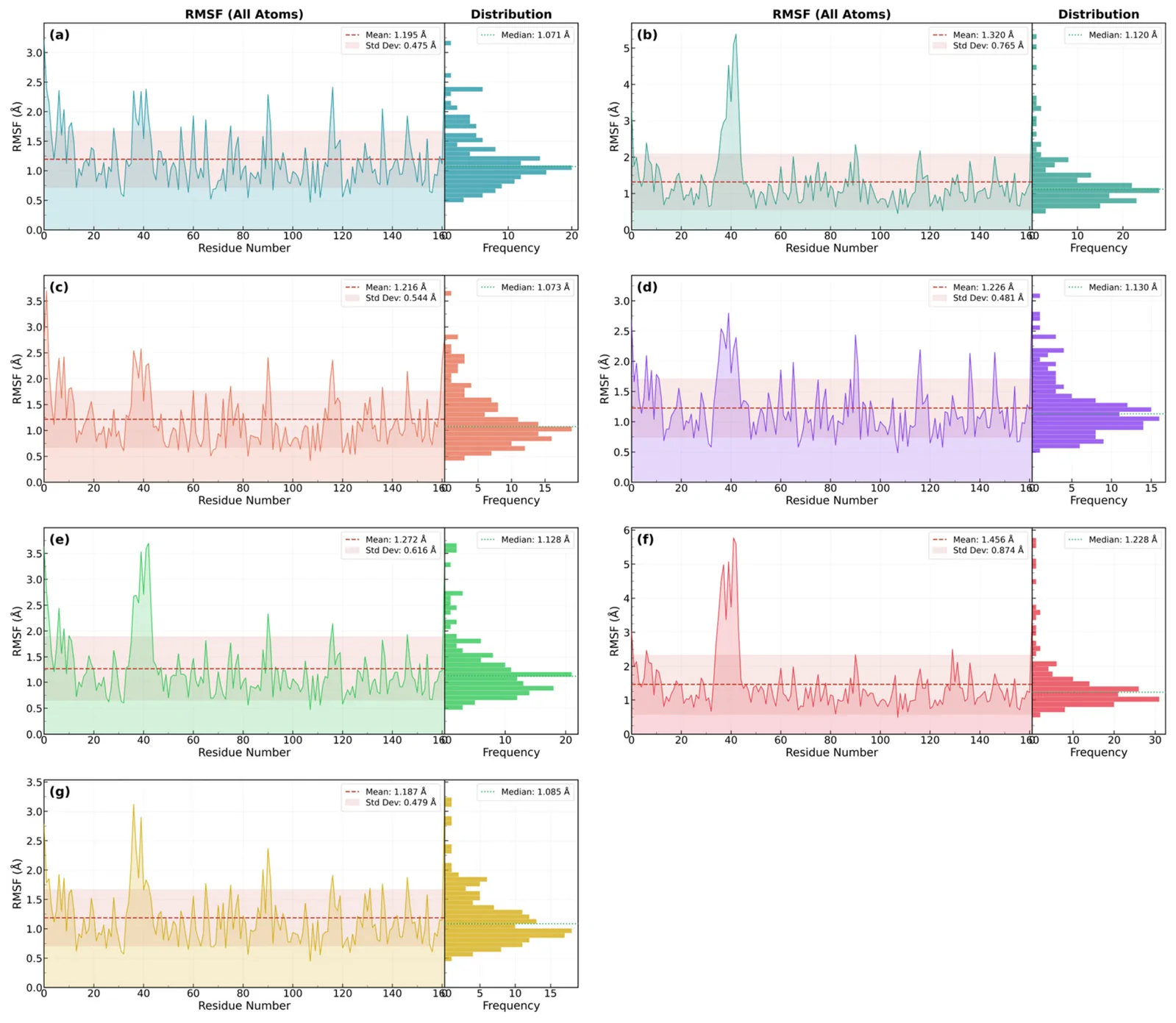
Residue flexibility analysis of the ligands bound to LasR in a dynamic environment. (**a**) shows the RMSF graph for the LasR-control complex with the mean RMSF 1.195 Å, (**b**) shows the RMSF graph for the LasR-DrugGPT M1 complex with the mean RMSF 1.522 Å, (**c**) shows the RMSF graph for the LasR-DrugGPT M2 complex with the mean RMSF 1.206 Å, (**d**) shows the RMSF graph for the LasR-DrugGPT M3 complex with the mean RMSF 1.376 Å, (**e**) shows the RMSF graph for the LasR-TD-1 complex with the mean RMSF 1.272 Å, (**f**) shows the RMSF graph for the LasR-TD-2 complex with the mean RMSF 1.456 Å, while (**g**) shows the RMSF for the apo protein.

RMSF analysis was used to determine atomic mobility in all seven complexes by calculating the per-residue flexibility. The LasR-control complex (Figure 7a) had an average RMSF of 1.195 ± 0.475 Å (median: 1.071 Å), with peaks in the inherently flexible loop and terminal regions. This complex yields a residue-level baseline dynamic for comparative study with the other systems. The LasR-DrugGPT M1 (Figure 7b) displayed elevated residue-level fluctuations relative to the control, particularly in the region spanning residues ~30–45, where a pronounced fluctuation peak exceeding 5 Å was observed associated with flexible loop regions adjacent to the binding cavity. The LasR-DrugGPT M2 shown in Figure 7c had a mean RMSF of 1.216 ± 0.544 Å (median: 1.073 Å), remaining highly comparable to the control system throughout most residue positions. Although several moderate peaks were observed and well controlled, indicating that LasR-DrugGPT M2 is largely preserved without inducing extensive conformational flexibility. Similarly, the LasR-DrugGPT M3 shown in Figure 7d demonstrated a mean RMSF value of 1.226 ± 0.481 Å (median RMSF = 1.130 Å), with residue fluctuations remaining relatively uniform across most of the protein structure. Higher flexibility was observed within the 30–45 residue region; however, these fluctuations were substantially lower than those observed in LasR-DrugGPT M1. On the other hand, the LasR-TD-1 complex (Figure 7e) demonstrated a mean RMSF value of 1.272 ± 0.616 Å (median RMSF = 1.128 Å) and had a distinctive peak of ~3.6 Å within residue 40, indicating an appreciable degree of localized flexibility in this region, possibly related to solvent-exposed loop structures.

Likewise, the LasR-TD-2 ligand-bound system shown in Figure 7f reported a similar level of flexibility around 35–50 and a significant residue-level heterogeneity in other regions with an RMSF of 1.456 ± 0.874 Å (median RMSF = 1.228 Å). Nevertheless, the elevated fluctuations remained largely localized rather than indicative of widespread destabilization of the overall protein structure. Finally, the apo LasR system shown in Figure 7g exhibited a mean RMSF of 1.187 ± 0.479 Å (median: 1.085 Å), displaying one of the most restrained fluctuation profiles among all systems. Although moderate peaks were observed near residues 35–45 and around residue 90, the overall residue-level dynamics remained highly controlled and comparable to the ligand-bound control complex. Collectively, these results show that the preserved native-like residue-level dynamic behavior relative to both the apo and control systems demonstrates modulation of local conformational dynamics rather than global structural destabilization of LasR. The highly dynamic regions during simulation are highlighted in the LasR structure and given in Supplementary Figure S1.

#### 2.4.4. Hydrogen Bond Analysis

**Figure 8:**
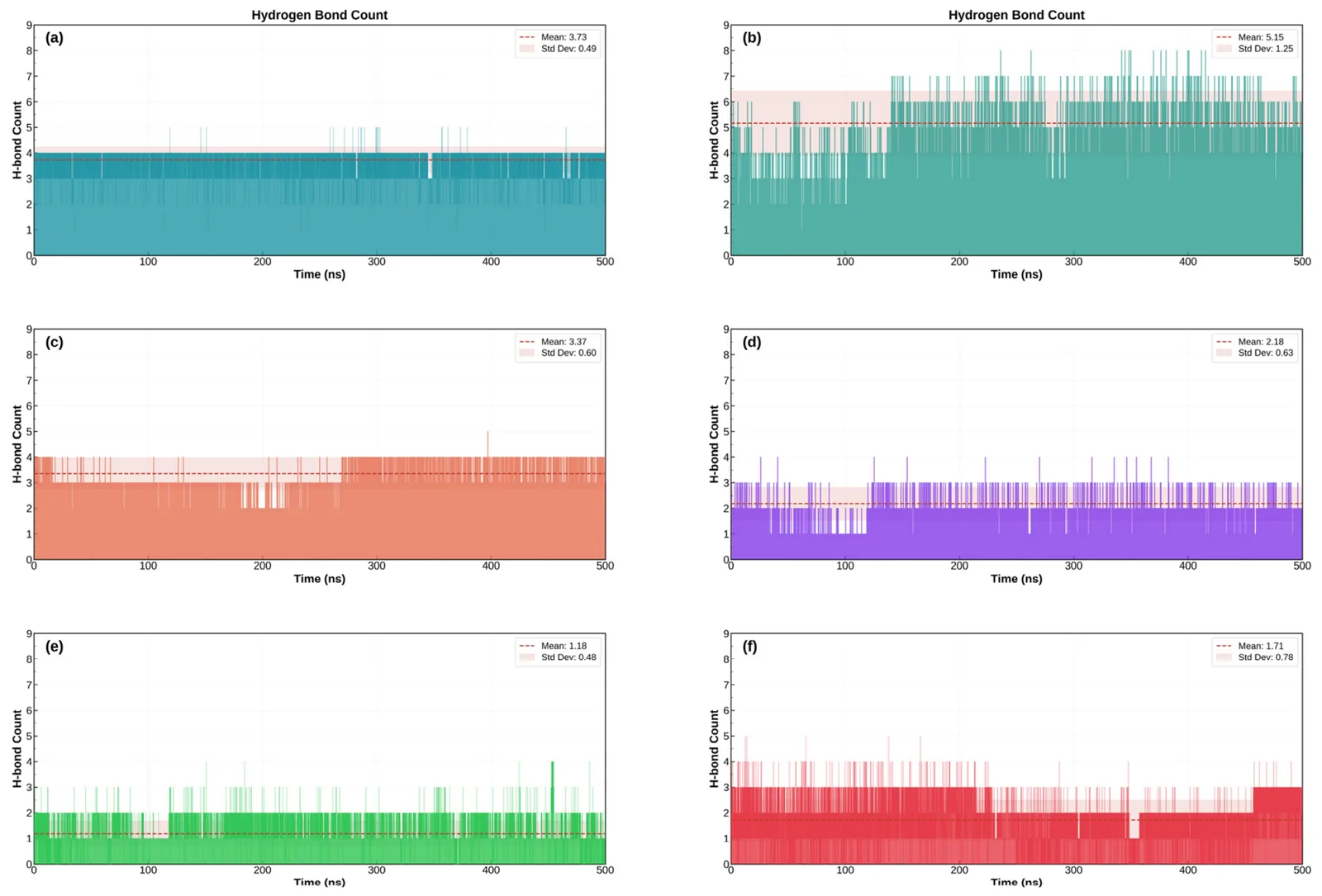
Time-resolved hydrogen bond analysis of LasR–ligand complexes over 500 ns MD simulations. The temporal evolution of hydrogen bond counts is shown for (**a**) control (6MWL), (**b**) LasR–DrugGPT M1, (**c**) LasR–DrugGPT M2, (**d**) LasR–DrugGPT M3, (**e**) LasR–TD-1, and (**f**) LasR–TD-2 complexes. The dashed red line represents the mean hydrogen bond count, and the shaded region shows the standard deviation, illustrating the extent of temporal fluctuations in intermolecular interactions across each system.

The number and stability of hydrogen bonds formed between each ligand and the protein were monitored throughout the 500 ns simulation to evaluate the polar interaction network sustaining each complex. The control system shown in Figure 8a maintained a stable hydrogen-bonding profile, with a mean of 3.73 ± 0.49 bonds per frame and a narrow distribution mainly centered around 3–4 hydrogen bonds, indicating a persistent polar interaction network within the binding site. In contrast, the LasR-DrugGPT M1 complex exhibited the highest average hydrogen-bond count (5.15 ± 1.23), as shown in Figure 8b. This system displayed markedly enhanced hydrogen-bond occupancy relative to the control, with bond numbers generally fluctuating between 4–7 throughout the simulation. Although the larger standard deviation suggests a more dynamic and adaptable hydrogen-bonding network, the overall interaction profile supports stronger polar stabilization. Similarly, LasR-DrugGPT M2 showed a hydrogen-bonding pattern comparable to the control system, with an average of 3.71 ± 0.49 bonds and a relatively stable distribution centered around 3–4 hydrogen bonds (Figure 8c).

Conversely, LasR-DrugGPT M3 exhibited a substantially lower average hydrogen-bond count (1.80 ± 0.75). Initially, the system maintained only 1–2 hydrogen bonds; however, after approximately 250 ns, a moderate increase in hydrogen-bond occupancy was observed, suggesting a progressive reorganization of ligand orientation within the binding cavity that resulted in a comparatively more stable, although weaker, polar interaction network (Figure 8d). Furthermore, LasR-TD-1 and TD-2 demonstrated the weakest hydrogen-bonding properties among all evaluated systems, with average bond counts of 1.18 ± 0.48 and 1.71 ± 0.78, respectively. Both systems showed sparse and intermittent hydrogen-bond formation during the simulation, particularly TD-1, which predominantly maintained only 0–2 hydrogen bonds with occasional transient increases. TD-2 exhibited slightly improved occupancy but still displayed substantial temporal fluctuations and inconsistent bond persistence (Figure 8e,f).

Overall, the triazole derivatives exhibited lower hydrogen-bond occupancy than the control and DrugGPT-derived complexes. However, hydrogen bonding alone does not fully explain ligand stability, as hydrophobic contacts, van der Waals interactions, aromatic packing, and shape complementarity within the LasR binding cavity also contribute substantially to stabilization. Thus, the triazole derivatives may rely more on hydrophobic-driven interactions, whereas DrugGPT M1 and M2 displayed the most persistent hydrogen-bonding profiles, supporting enhanced polar stabilization throughout the simulation.

#### 2.4.5. Hydrogen Bond Geometry and Occupancy Analysis

The hydrogen-bond geometric properties of all six protein–ligand complexes were evaluated using donor–acceptor (D···A) distance and donor–hydrogen–acceptor (D–H–A) angle distributions extracted from the 500 ns trajectories. The control system (6MWL) exhibited the most favorable hydrogen-bond geometry, with the shortest mean donor–acceptor distance (2.93 Å) and the most linear mean D–H–A angle (161.0°). Among the designed compounds, DrugGPT M3 most closely approximated the control geometry, particularly in donor–acceptor distance (2.94 Å), whereas DrugGPT M2 demonstrated highly favorable angular linearity (160.8°). DrugGPT M1 occupied an intermediate geometric position, while TD-1 and especially TD-2 exhibited progressively weaker geometries characterized by longer donor–acceptor distances and reduced angular linearity. These geometric deviations were consistent with the reduced hydrogen-bond persistence observed previously for TD-1 and TD-2. The bonding geometries are shown in Supplementary Figure S2a–f.

Hydrogen-bond occupancy analysis across the 500 ns trajectories further revealed distinct interaction architectures among the complexes. The control complex exhibited a highly persistent interaction network dominated by Asp67 (99.4%), Tyr50 (96.3%), Ser123 (88.1%), and Trp54 (82.3%). DrugGPT M1 demonstrated the most spatially distributed occupancy profile among the designed ligands, involving highly persistent interactions with Trp54, Tyr87, Asp59, and Arg55, suggesting adaptive ligand positioning and extensive interaction plasticity within the binding cavity. In contrast, DrugGPT M2 displayed the most balanced and structurally coherent interaction network, strongly centered on Asp67, Ser123, Trp54, and Tyr50, closely resembling the control system and supporting its previously observed conformational stability. DrugGPT M3 formed a more localized interaction network dominated primarily by Asp67 anchoring with fewer persistent secondary contacts. Conversely, TD-1 exhibited a highly restricted interaction profile mainly involving Thr109, with weak secondary interactions and absence of persistent Asp67 or Tyr50 engagement, indicating a distinct and less integrated binding orientation.

TD-2 partially restored Asp67 anchoring but retained a fragmented and spatially dispersed interaction network lacking the coherent multi-residue stabilization observed for DrugGPT M2 and the control complex. Overall, Asp67 served as the principal anchoring residue across nearly all systems except TD-1, whereas Ser123, Tyr50, and Trp54 contributed important secondary stabilization. Collectively, the occupancy heatmap supports DrugGPT M2 as the most stable and structurally coherent binding mode among the designed ligands, while TD-1 and TD-2 exhibited comparatively weaker and less integrated polar interaction architectures. The hydrogen-bond occupancy heatmaps are shown in Supplementary Figure S3.

#### 2.4.6. MM-GBSA Binding Free Energy Analysis

The MM-GBSA method was used to calculate binding free energies (ΔG_bind_) and individual energy components for all six of the complexes over the final 100 ns of their respective trajectories. The results reported in Table 2 are presented as the mean ± standard deviation (kcal/mol) of the total binding free energies. The results demonstrate a clear hierarchy of binding strength across the six systems. Among these, the LasR–DrugGPT M3 reported the strongest total binding free energy of −102.76 ± 5.41 kcal/mol, while the LasR–DrugGPT M1 complex was ranked second among the six by demonstrating an overall binding energy of −102.32 ± 6.25 kcal/mol. Moreover, the total binding energy for LasR–DrugGPT M2 was reported to be −99.92 ± 4.24 kcal/mol, for LasR-TD-1 −96.81 ± 5.71 kcal/mol, and for LasR-TD-2 −89.30 ± 4.26 kcal/mol, while the control complex reported a total binding energy of −80.43 ± 4.99 kcal/mol. Accordingly, the rank order of total binding affinity is: LasR–DrugGPT M3, LasR–DrugGPT M1, LasR–DrugGPT M2, LasR–TD-1, LasR–TD-2, and LasR-control. Among the six complexes, all designed ligands exhibited more favorable binding free energies than the control system, with LasR–DrugGPT M3 showing the largest improvement.

The vdW term, which reflects shape complementarity and hydrophobic packing within the binding pocket, is the most consistently stabilizing component across all systems. The vdW contribution for all binding complexes revealed LasR–DrugGPT M3 −63.66 ± 3.13 kcal/mol, LasR–TD-1 −62.94 ± 3.44 kcal/mol, LasR–DrugGPT M2 −60.03 ± 2.53 kcal/mol, LasR–DrugGPT M1 −57.58 ± 3.59 kcal/mol, LasR–TD-2 −52.72 ± 3.36 kcal/mol, and LasR-control −50.04 ± 2.71 kcal/mol, respectively. LasR–DrugGPT M3, LasR–TD-1, and LasR–DrugGPT M2 exhibit particularly strong vdW interactions, indicating excellent steric complementarity and hydrophobic accommodation within the LasR binding cavity. Unlike hydrogen-bond occupancy trends, the MM-GBSA results show that binding stabilization in several complexes is driven predominantly by van der Waals and lipophilic interactions rather than by hydrogen bonding alone. This behavior is particularly evident for LasR–DrugGPT M3 and LasR–TD-1, where highly favorable vdW and lipophilic interactions compensate for comparatively reduced hydrogen-bond persistence, resulting in strongly favorable overall binding free energies. LasR–TD-2 exhibited the strongest Coulombic contribution (−89.36 ± 4.73 kcal/mol), which is consistent with the strong hydrogen-bond occupancy involving Asp67 observed in the occupancy analysis. LasR–DrugGPT M1 also demonstrated substantial electrostatic stabilization (−47.64 ± 5.66 kcal/mol) together with a highly distributed hydrogen-bond interaction network. In contrast, LasR–TD-1 exhibited the weakest Coulombic contribution (−16.75 ± 3.40 kcal/mol), confirming that its binding mechanism relies predominantly on hydrophobic packing and lipophilic stabilization rather than polar interactions, consistent with its relatively low hydrogen-bond occupancy.

The large SolvGB (solvation/desolvation) penalty for LasR–TD-2 (81.98 ± 4.27 kcal/mol) mirrors its strong Coulombic interaction contribution, since highly charged or polar interactions require substantial desolvation before complex formation. In contrast, the DrugGPT-derived ligands exhibited a more balanced energetic decomposition profile involving simultaneous vdW, lipophilic, and electrostatic stabilization components. Differential binding affinities among the various LasR–DrugGPT and LasR–TD compounds were confirmed by MMGBSA, with LasR–DrugGPT M3 exhibiting the most favorable ΔGbind (−102.76 ± 5.41 kcal/mol), primarily driven by exceptionally strong vdW and lipophilic stabilization. LasR–DrugGPT M1 (−102.32 ± 6.25 kcal/mol) similarly demonstrated highly favorable binding, driven by a combination of electrostatic complementarity and an extensive multi-residue hydrogen-bond interaction network. LasR–DrugGPT M2 (−99.92 ± 4.24 kcal/mol) exhibited the most geometrically coherent interaction profile, supported by balanced vdW and persistent polar interactions centered around Asp67, Ser123, Tyr50, and Trp54. LasR–TD-1 achieved strong binding predominantly through hydrophobic and lipophilic interactions, despite limited hydrogen-bond persistence, whereas LasR–TD-2 relied heavily on electrostatic anchoring interactions involving Asp67. The MM/GBSA calculations resulted in the binding free energies ranging from −80.43 to −102.76 kcal/mol. Although these values are more favorable than experimentally derived free energies typically reported for small-molecule inhibitors, such behavior is commonly observed in MM/GBSA calculations due to methodological approximations, including incomplete treatment of conformational entropy and the use of implicit solvation models. Consequently, the primary value of MM/GBSA lies in its ability to provide internally consistent relative binding estimates across a ligand series rather than absolute predictions of experimentally measured binding free energies. Instead, the approach is most valuable for comparative ranking of ligands evaluated under identical simulation conditions. In the present study, the superior binding affinities observed for DrugGPT M1, DrugGPT M2, DrugGPT M3, and Triazole-1 were consistently supported by favorable van der Waals, electrostatic, and lipophilic interaction energies, indicating stable and energetically favorable protein–ligand complexes throughout the simulation trajectories. Large binding free energies have also been reported elsewhere, corroborating our findings [24].

#### 2.4.7. Principal Component Analysis of Conformational Dynamics

Principal component analysis (PCA) was performed on the Cα backbone coordinates of all six protein–ligand complexes over the 500 ns simulations to evaluate conformational sampling and ligand-induced dynamic behavior relative to the reference system. The control complex (6MWL; LasR-control) displayed a compact and well-converged conformational distribution, with PC1 and PC2 accounting for 20.41% and 17.95% of the total variance, respectively (combined variance: 38.36%). The limited conformational spread and modest trajectory drift indicate a stable and conformationally restricted complex throughout the simulation. In contrast, LasR–DrugGPT M1 exhibited a substantially broader conformational distribution, with PC1 and PC2 explaining 61.38% and 8.27% of the variance, respectively (combined variance: 69.65%). The PCA scatter plot revealed multiple partially overlapping conformational clusters, particularly along PC1, suggesting enhanced ligand-induced conformational plasticity and sampling of several metastable substates.

LasR–DrugGPT M2 demonstrated a multi-basin conformational profile, with PC1 and PC2 accounting for 30.33% and 21.09% of the variance (combined variance: 51.42%). The presence of several moderately overlapping clusters indicates stable transitions between multiple conformational substates while preserving overall structural integrity. Similarly, LasR–DrugGPT M3 exhibited an intermediate conformational regime with broad but continuous sampling and strong overlap between early and late simulation frames, supporting stable ligand binding with moderate flexibility and minimal structural destabilization. In contrast, LasR-TD-1 showed broader sampling and moderate conformational drift, whereas LasR-TD-2 exhibited the highest conformational dispersion among all systems. For TD-2, PC1 alone accounted for 50.62% of the variance, with combined PC1 + PC2 variance reaching 70.38%, accompanied by a pronounced directional separation between early and late simulation frames, indicating persistent ligand-induced conformational transitions and increased structural mobility.

**Figure 9:**
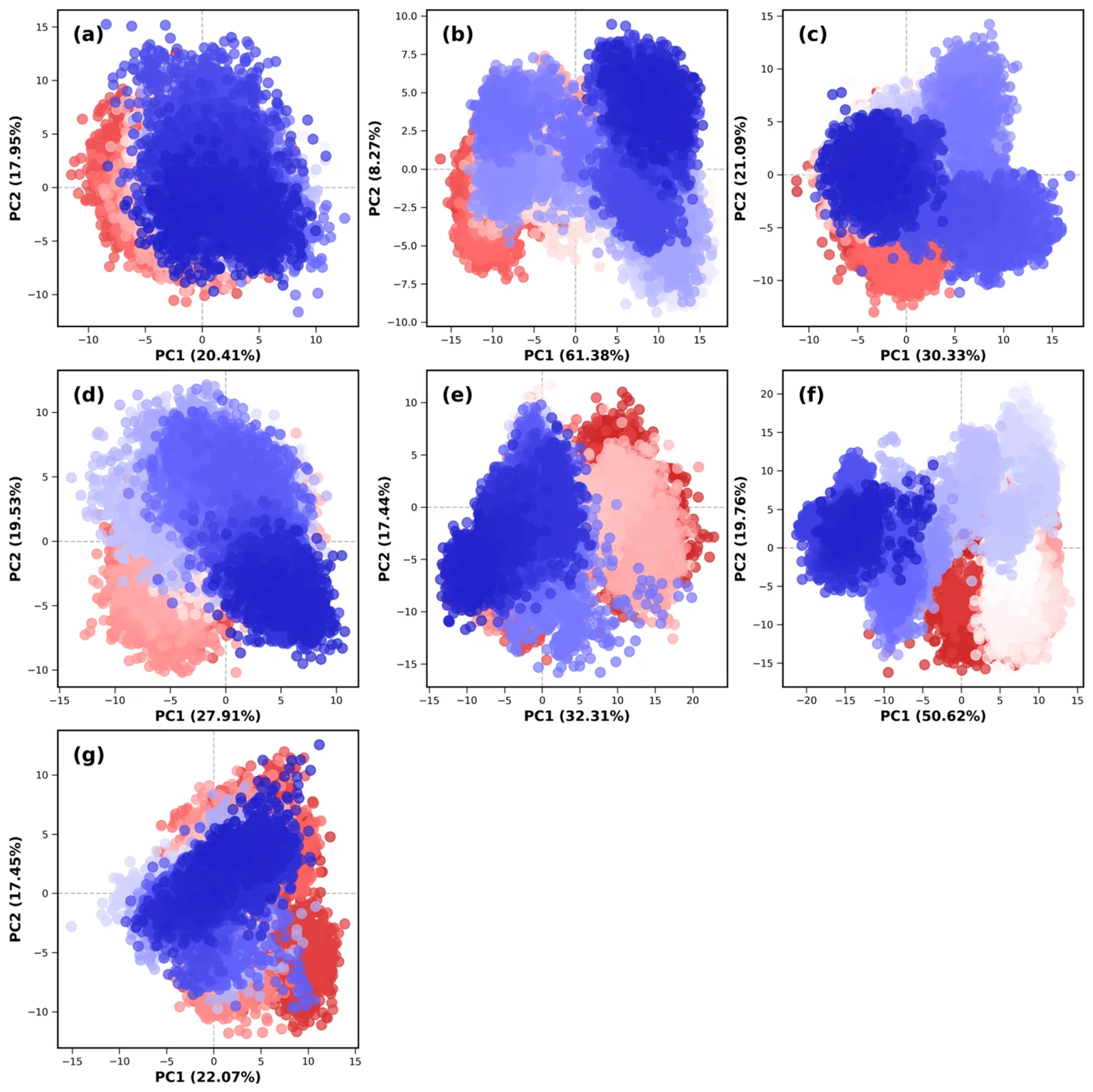
Principal component analysis (PCA) for each complex. The PCA for each complex is shown for (**a**) LasR-control (6MWL), (**b**) LasR–DrugGPT M1, (**c**) LasR–DrugGPT M2, (**d**) LasR–DrugGPT M3, (**e**) LasR–TD-1, (**f**) LasR–TD-2 complexes, and (**g**) shows the PCA graph over the simulation time for the apo protein. The red color represents the start of the simulation (0 ns), with the pattern changing to blue (end of simulation).

The apo LasR system displayed a relatively compact and diagonally distributed conformational landscape, with PC1 and PC2 accounting for 22.07% and 17.45% of the variance, respectively (combined variance: 39.52%). Considerable overlap between early and late frames suggests stable intrinsic protein dynamics in the absence of ligand binding. Overall, the PCA establishes the following hierarchy of conformational stability: LasR-control ≈ apo LasR > DrugGPT M3 > DrugGPT M2 > TD-1 > DrugGPT M1 > TD-2. Systems exhibiting compact or continuously overlapping conformational distributions, particularly the control, apo, and DrugGPT M3 complexes, are most consistent with stable and dynamically coherent behavior, whereas TD-2 and DrugGPT M1 demonstrate increased conformational plasticity and ligand-induced collective motion. The PCA plots are shown in Figure 9a–g.

#### 2.4.8. Free Energy Landscape Analysis

The free energy landscapes (FELs) generated from the PC1–PC2 conformational space revealed distinct thermodynamic behaviors among all complexes and the apo system. The control complex (LasR-control) displayed a single deep and tightly confined energy basin, indicating a highly stable and conformationally restricted binding mode throughout the simulation. In contrast, LasR–DrugGPT M1 exhibited a broader and more heterogeneous landscape composed of two partially connected low-energy basins separated by a shallow energetic barrier, suggesting dynamic interconversion between metastable conformations and increased ligand-induced conformational adaptability. LasR–DrugGPT M2 demonstrated a comparatively stable and thermodynamically favorable topology characterized by a dominant low-energy basin connected to several shallow subsidiary minima, supporting stable equilibration without major energetic barriers. Similarly, LasR–DrugGPT M3 exhibited a relatively compact free-energy landscape with a central dominant basin and a few neighboring minima, indicating stable conformational sampling with limited large-scale structural transitions.

Conversely, LasR-TD-1 displayed a more rugged and asymmetrically distributed free-energy surface with several shallow minima dispersed across a broader conformational region, reflecting increased structural heterogeneity relative to the control and DrugGPT-derived systems. LasR-TD-2 showed the most spatially dispersed and thermodynamically heterogeneous landscape among all ligand-bound systems, with multiple separated low-energy minima distributed across a wide conformational space, consistent with the extensive conformational plasticity observed in the PCA, RMSD, and RMSF analyses. The apo LasR system exhibited a moderately compact but broader free-energy landscape than the ligand-bound control complex, reflecting the greater intrinsic flexibility of the unbound receptor.

**Figure 10:**
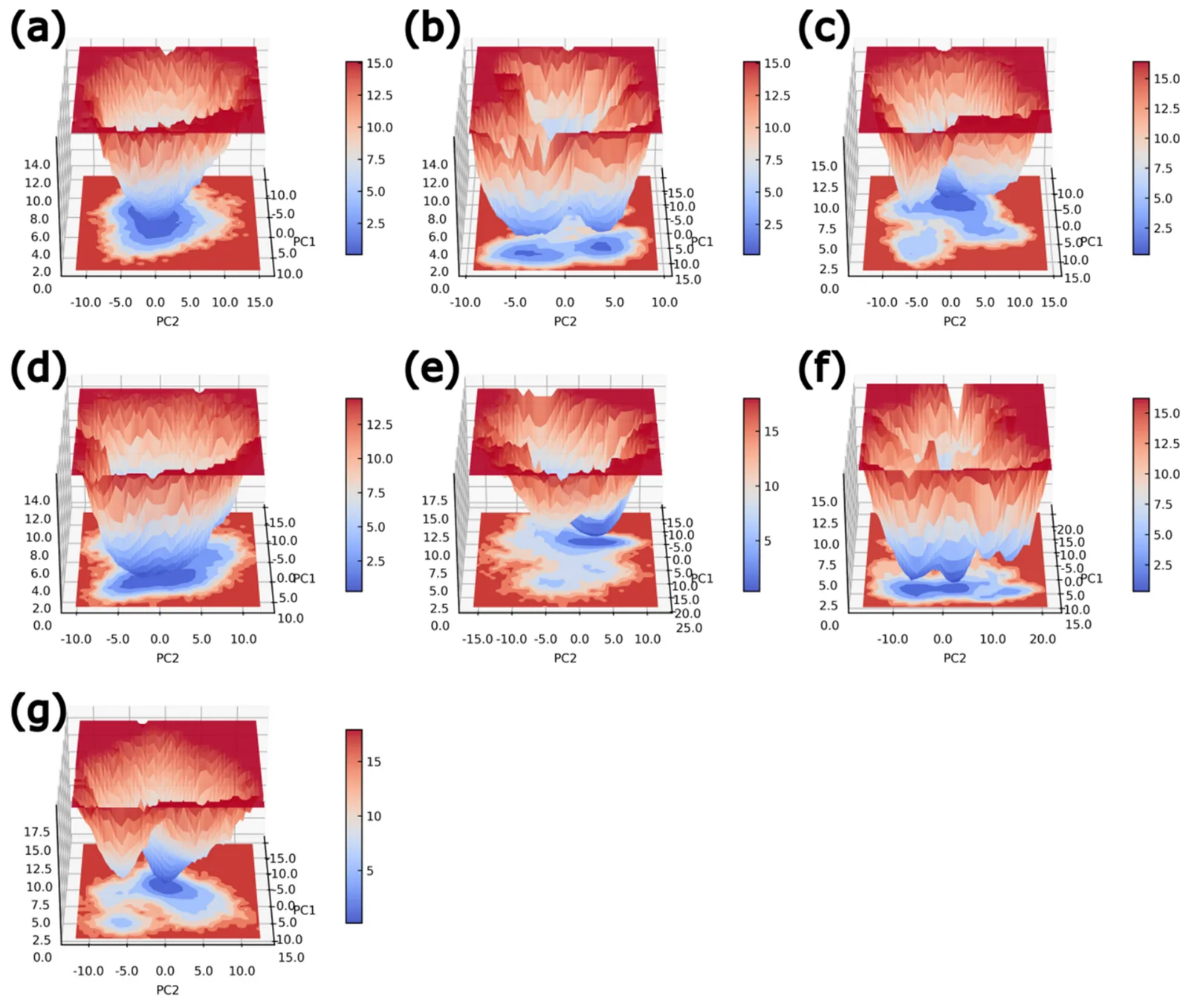
Free Energy Landscape (FEL) analysis for each complex. The FEL for each complex is shown for (**a**) LasR-control (6MWL), (**b**) LasR–DrugGPT M1, (**c**) LasR–DrugGPT M2, (**d**) LasR–DrugGPT M3, (**e**) LasR–TD-1, (**f**) LasR–TD-2 complexes, and (**g**) shows the FEL graph over the simulation time for the apo protein.

Overall, the FEL analysis demonstrates clear ligand-dependent modulation of LasR conformational dynamics. Among the designed ligands, DrugGPT M2 and DrugGPT M3 exhibited the most thermodynamically favorable and conformationally stable landscapes, whereas DrugGPT M1 and particularly TD-2 displayed increased energetic dispersion and conformational heterogeneity. Collectively, integration of the FEL, MM-GBSA, hydrogen-bond occupancy, RMSD, RMSF, Rg, and PCA analyses supports DrugGPT M2 and DrugGPT M3 as the most stable and promising candidates for further experimental validation against LasR. The FEL plots for all complexes are shown in Figure 10a–g.

## 3. Materials and Methods

### 3.1. Target Structure Retrieval and Preparation

The three-dimensional structure of the LasR ligand-binding domain from *P. aeruginosa* was obtained from the Protein Data Bank (PDB) with the PDB ID: 6MWL [25]. The co-crystallized structure with the autoinducer ligand was selected to determine the binding pocket of the LasR receptor accurately. Protein preparation was performed using the Protein Preparation Wizard implemented in Schrödinger Maestro (Schrödinger Release 2023-4). Bond orders were assigned, missing hydrogen atoms were added, and side chains or any missing residues were reconstructed as necessary. The protonation state of any ionizable residues was predicted using PROPKA at physiological pH (7.0) [26,27]. An optimal hydrogen bonding network was created, and any crystallographic water molecules located more than 5 Å from the ligand binding site were removed. Finally, the structure was minimized using the OPLS4 force field to achieve restrained heavy-atom convergence at an RMSD of 0.3 Å [28]. This minimization was performed to remove steric clashes while maintaining the integrity of the original structure, as determined by experimental data.

### 3.2. Docking Protocol Validation

The docking protocol was validated by redocking the co-crystallized ligand, *4-(3-bromophenoxy)-N-[(3S)-2-oxothiolan-3-yl]butanamide*, into the prepared binding pocket, and the RMSD was calculated between the docked pose and the experimental. Before docking, the ligand was extracted and prepared using LigPrep (Schrödinger Release 2023-4) and docked using the Glide XP module (Schrödinger Release 2023-4) [29].

### 3.3. Triazole-Based Substructure Search and Compound Retrieval

Triazole scaffolds were selected for their known antimicrobial activity, metabolic stability, and strong hydrogen-bonding capability. A substructure-based search was performed in the PubChem database using both *1,2,3-triazole* and *1,2,4-triazole* motifs to retrieve a set of substructures for screening based on the given scaffolds [30]. The basic scaffold SMILES of triazole were used as input queries using default parameters and substructure criteria to retrieve all the relevant compounds that share this moiety.

### 3.4. De Novo Drug Design Using DrugGPT

To expand the chemical diversity beyond existing database compounds, additional molecules were generated using DrugGPT (https://github.com/LIYUESEN/druggpt.git, accessed on 29 May 2026), a transformer-based generative model for molecular design. DrugGPT employs a generative pre-trained transformer architecture that treats chemical structures as sequential representations encoded in SMILES format [31]. The model consisted of 12 transformer layers, 12 multi-head self-attention heads, and a hidden state dimension of 768, enabling it to capture long-range dependencies in molecular sequences. The model had previously been pretrained on large chemical datasets, including ChEMBL and ZINC, enabling it to learn chemical grammar and structural patterns within drug-like chemical space. During generation, the model produced new molecules autoregressively using top-k sampling (k = 50) and nucleus sampling (*p* = 0.95) to balance structural diversity and chemical validity. The generated SMILES strings were filtered to remove chemically invalid structures, thereby avoiding the prioritization of invalid molecules. Ligand SMILES were generated in batches using stochastic sampling with top-k, top-p, and temperature control. Generated SMILES were validated with Open Babel, filtered to exclude non-hydrogen atoms, converted to 3D SDFs using MMFF94, and invalid or empty SDFs were removed. Duplicate tracking was performed by SHA-1 hashing ligand SMILES, and the final ligands were subjected to parallel MMFF94 energy minimization using OpenBabel with multiprocessing.

### 3.5. Ligand Library Preparation

Before screening both triazole-based and our *de novo*-designed compounds, we prepared the libraries using the LigPrep module in Schrödinger Maestro. The preparation involved removing the salts, duplicates, and incomplete structures and minimizing each molecule using the MMFF94 force field. Moreover, possible protonation states, tautomers, and stereoisomers at physiological conditions (pH 7.0 ± 2.0) and optimized ligands were obtained and subjected to Lipinski’s rule of five. The final ligand library consisted of both PubChem-derived triazole compounds and DrugGPT-generated derivatives, which were filtered and prepared for screening, enabling exploration of both known and novel chemical space.

### 3.6. Virtual Screening Using the Glide Scoring Function

Virtual screening of the curated ligand library was performed using the Glide virtual screening module in Schrödinger Maestro (Schrödinger Release 2023-4) [32]. The receptor grid was generated around the ligand-binding domain of LasR, with the co-crystallized autoinducer coordinates used as the grid center to ensure accurate representation of the native binding pocket. The grid box dimensions were adjusted to encompass the entire active site region, allowing sufficient conformational freedom for ligand placement. A hierarchical three-step docking workflow was implemented, consisting of High-Throughput Virtual Screening (HTVS), Standard Precision (SP), and Extra Precision (XP) docking stages [32]. In the first step, HTVS compounds with poor steric and electrostatic complementarity were rapidly removed, and the best-scoring compounds were retained and redocked using the SP mode to improve docking accuracy. Thereafter, a more stringent and accurate criterion, XP docking, was used to rank and shortlist the top 10% of the total compounds against LasR. The GlideScore, an empirical scoring function derived from ChemScore and Glide-specific terms, was used to estimate ligand binding affinity. This scoring function incorporates contributions from van der Waals, electrostatic, hydrogen-bonding, lipophilic-contact, desolvation, and ligand-strain energies. The top-ranking compounds with the most favorable Glide XP scores were selected as candidate hits for further high-accuracy docking and molecular simulation investigation.

### 3.7. Molecular Redocking Using GNINA

To obtain more accurate binding pose predictions and incorporate machine-learning-based scoring, the optimized ligands were redocked using GNINA (version 1.3) (https://github.com/gnina/gnina.git, accessed on 29 May 2026) [33]. GNINA extends the AutoDock Vina docking (1.2.0) framework by integrating three-dimensional convolutional neural networks (3D-CNNs) for protein–ligand scoring and pose evaluation [34]. The receptor and ligands were converted to PDBQT format using AutoDockTools, and docking was performed within the validated LasR ligand-binding region identified during receptor preparation [35]. Conformational samples of a ligand are generated by random rotation, translation, and torsional angling of rotatable bonds of the ligand’s structure. The generated conformational samples are then subjected to energy minimization using the grid. The exhaustiveness parameter determines the number of independent search operations performed during a docking experiment. This neural network architecture has been trained on experimentally determined protein–ligand interactions contained in the PDBBind database. The representation of the ligand and protein–ligand complex utilized in the CNN is a three-dimensional grid of voxels containing the atomic density values and chemical characteristics of the ligand and its corresponding protein receptor. Each voxel representation is passed through multiple convolutional layers to capture the spatial interaction patterns of the protein–ligand complex, including hydrogen bonding, hydrophobic interactions, and steric complementarity. In the end, the CNN produces two important outputs for each evaluated pose: the quality of the docking pose, as measured by the CNN Pose Score, and the predicted binding affinity, as measured by the CNN Affinity Score. The CNN score values are then used during docking to rescore and improve docking poses generated with the classical PDB Vina scoring model. The most favorable poses were selected based on consensus scoring and visual inspection of key interactions with residues within the LasR ligand-binding pocket.

### 3.8. Molecular Dynamics Simulation

Molecular dynamics (MD) simulations were performed to investigate the structural stability and dynamic behavior of the LasR–ligand complexes using the Desmond simulation engine in Schrödinger Suite (Release 2023-3) [36]. System preparation and simulation workflows were automated using the AutoMD pipeline, which integrates system construction, equilibration, and production simulations within the Desmond multisim framework (https://github.com/Wang-Lin-boop/AutoMD, accessed on 29 May 2026). The protein–ligand complexes obtained from molecular docking were imported into Maestro (.mae format) and prepared using the System Builder module. Each system was solvated with the SPC explicit water model in a cubic periodic boundary box, with a 15 Å buffer between the solute and the box boundary. System neutrality was achieved by adding K^+^ counterions, and 0.15 M KCl was introduced to mimic physiological ionic strength. The S-OPLS force field was used to parameterize both protein and ligand atoms. Energy minimization and equilibration were performed using an automated protocol that included Brownian and Langevin dynamics stages. Initially, two 100 ps Brownian dynamics simulations under the NVT ensemble at 10 K were performed, with positional restraints on the solute heavy atoms to relax the system. Brownian dynamics was used because it provides controlled damping of atomic motions in the initial equilibration phase, allowing the system to approach a physically stable configuration before full classical molecular dynamics equilibration. Following this initial low-temperature relaxation step, the systems were gradually heated to 300 K and subsequently equilibrated under standard MD conditions before the production simulations were performed. This was followed by short Langevin dynamics equilibration stages, including 12 ps NVT and NPT simulations, during which restraints were gradually released to stabilize the system. Production MD simulations were then performed for 500 ns under the NPT ensemble using a Langevin thermostat and Martyna–Tobias–Klein (MTK) barostat, maintaining a temperature of 310 K and pressure of 1.01325 bar [37]. A 9 Å cutoff was used for short-range nonbonded interactions, while long-range electrostatics were treated using the Particle Mesh Ewald (PME) method [38]. The integration timestep was set to 2 fs for bonded interactions and 6 fs for long-range interactions. Trajectory frames were recorded at 20 ps intervals, producing approximately 25,000 frames over the 500 ns simulation, and were stored in Desmond DTR format for subsequent structural and energetic analyses. Stability and binding behavior of the complexes were evaluated using root-mean-square deviation (RMSD), root-mean-square fluctuation (RMSF), hydrogen-bond occupancy, and protein–ligand interaction profiling.

### 3.9. Post-Simulation Trajectory Analysis

After completing the molecular simulations, the resulting trajectories were analyzed using an in-house Python script to evaluate the stability and interaction behavior of the protein–ligand complexes. Structural stability via RMSD, folding via Rg, residue flexibility via RMSF, hydrogen bonding, H-bond distances and angles, and occupancy were assessed. To further evaluate interaction persistence, ligand contact frequency analysis was performed on equilibrated trajectory frames, with contacts defined using a 4.0 Å cutoff between ligand heavy atoms and protein residues. Contact frequencies were calculated based on the number of frames in which residues maintained contacts with the ligand relative to the total number of analyzed frames, allowing identification of key residues responsible for stabilizing the ligand within the LasR binding pocket and characterization of dominant interaction hotspots during the simulation.

### 3.10. Binding Free Energy Calculations

To estimate the thermodynamics-based binding stability of the protein–ligand complexes, Molecular Mechanics Generalized Born Surface Area (MM-GBSA) calculations were performed on representative trajectory frames extracted from the equilibrated region of the simulation [39]. The binding free energy shown in Equation (1) was calculated, and the energetic contributions described in Equation (2), including bonded, electrostatic, van der Waals, polar solvation, and non-polar solvation terms.

Mathematically, the binding free energy can be estimated as:

(1) ΔG(bind) = ΔG(complex) − [ΔG(receptor) + ΔG(ligand)]

The following equation calculated the different contributing components of total binding energy:

(2) G=Gbond + Gele +GvdW +Gpol +Gnpol

where

**Gbond**: Bonded interaction energy, including contributions from bond stretching, angle bending, and dihedral (torsional) terms.
**Gele**: Electrostatic energy arising from Coulombic interactions between charged atoms.
**GvdW**: Van der Waals energy, representing non-bonded interactions such as dispersion and steric repulsion.
**Gpol**: Polar solvation energy, typically calculated using continuum electrostatics methods such as Poisson–Boltzmann or Generalized Born models.
**Gnpol**: Non-polar solvation energy, associated with hydrophobic effects and solvent-accessible surface area (SASA).
Multiple trajectory snapshots from the last 100 ns of the production simulation were used to estimate binding free energy and ensure convergence.

### 3.11. Principal Component Analysis (PCA)

Principal component analysis (PCA) [40] was performed on the Cα backbone atoms extracted from the 500 ns molecular dynamics trajectories of all LasR–ligand complexes and the apo system to characterize dominant collective motions and conformational sampling behavior. Before analysis, all trajectory frames were structurally aligned to the reference starting structure to remove global translational and rotational motions. The covariance matrix of atomic positional fluctuations was then calculated using Equation (3), followed by diagonalization of the covariance matrix using Equation (4) to obtain the corresponding eigenvectors and eigenvalues. The eigenvectors associated with the largest eigenvalues define the principal components that describe the dominant collective motions observed during the simulations. The first two principal components (PC1 and PC2) were projected and visualized as 2D conformational scatter plots to assess conformational drift, clustering behavior, and sampling convergence across trajectories. Frames were colored progressively from red (0 ns) to blue (500 ns) to monitor the temporal evolution of conformational sampling. The PC1–PC2 projections were also used to construct free energy landscapes (FELs) based on Boltzmann population distributions to identify low-energy conformational basins and ligand-induced thermodynamic changes in LasR dynamics. When given a collection of n-dimensional vectors (xi), the covariance matrix (C) can be calculated using the following mathematical representation:

(3) $$C=\;\frac{1}{N}\;\times \;\sum \left(i=1\;to\;N\right)\left[\;{(x}_{i}-\;\mu \right)\;\times \;(x_{i}-\;\mu {)}^{T}]$$

where

- N = number of vectors;
- μ = mean vector;
- T designates the transpose operation.

The following equation can be used to obtain the eigenvectors (V) and eigenvalues (λ) of the covariance matrix C.

(4) C×V=λ×V

where V stands for the eigenvector matrix and λ for the diagonal matrix of eigenvalues. The principal components of the system are represented by the eigenvectors with the highest matching eigenvalues.

### 3.12. Free Energy Landscape (FEL)

The conformational dynamics and thermodynamic behavior of the protein–ligand complexes were further characterized by constructing free-energy landscapes (FELs) based on the first two principal components derived from PCA [41]. The free energy surface was estimated using the Boltzmann distribution:

(5) G(x,y) = −k_B T \ln P(x,y)

where

- k_B and T are the Boltzmann constant and the absolute temperature.

The probability distribution P(x, y) was obtained from the population density of MD trajectory frames, projected onto PC1 and PC2 via PCA. The resulting FEL describes the thermodynamically preferred conformational states sampled during simulation.

## 4. Conclusions

This study demonstrates that integrating triazole scaffold repurposing with transformer-based generative modeling enables the efficient discovery of high-affinity LasR inhibitors targeting quorum sensing in *Pseudomonas aeruginosa*. The identified lead compounds exhibited significantly improved docking scores (up to −11.83 kcal/mol), stable MD behavior (RMSD ~2.2–3.0 Å), and favorable binding energetics, highlighting their potential as anti-virulence therapeutics. The consistency of key residue interactions (Tyr50, Asp67, Ser123) further supports their mechanistic relevance. Importantly, this AI-driven pipeline provides a scalable and reproducible framework for exploring chemical space beyond traditional libraries. Although independent replicate MD simulations were not performed due to the high computational cost associated with multiple 500 ns production runs, the reliability of the results was strengthened through long-timescale simulations, ensemble-based MM-GBSA calculations using multiple equilibrated trajectory frames, and multiple orthogonal trajectory analyses, including RMSD, RMSF, Rg, hydrogen-bond occupancy, PCA, and free-energy landscape analysis. Additionally, the absence of in vitro and in vivo validation restricts immediate translational applicability. Future work should focus on experimental validation, ADMET profiling, and optimization of pharmacokinetic properties to advance these candidates toward clinical development.

## Figures and Tables

**Table 1:** The table shows the best hits identified from a three-step virtual screening protocol applied to triazole scaffolds and to novel target-aware de novo compounds. The table also shows the Names, XP docking scores, GNINA Scores, and drug-likeness status.

Compounds Names	XP Docking Score (kcal/mol)	GNINA Score	Drug-Likeness (Lipinski)
**Triazole 1**	−11.70	−9.211	Passed
**Triazole 2**	−11.59	−8.746	Passed
**DrugGPT M1**	−13.81	−10.479	Passed
**DrugGPT M2**	−12.42	−9.902	Passed
**DrugGPT M3**	−12.11	−9.721	Passed
**Control**	−8.50	−6.667	Passed

**Table 2:** MM-GBSA binding free energy decomposition for LasR–ligand complexes over the 500 ns MD simulations. Reported values represent mean ± standard deviation (kcal/mol) for the total binding free energy (ΔG_bind_) and its individual energetic components, including Coulombic (electrostatic), van der Waals (vdW), lipophilic, hydrogen-bond, and polar solvation (Solv_GB_) contributions. Ligand efficiency values are also provided to assess binding performance relative to ligand size.

Systems	ΔG_Bind_ (kcal/mol)	Coulomb (kcal/mol)	vdW (kcal/mol)	Lipophilic (kcal/mol)	H-Bond (kcal/mol)	Solv_GB_ (kcal/mol)	Ligand Efficiency
**Control**	−80.43 ± 4.99	−28.07 ± 3.49	−50.04 ± 2.71	−21.93 ± 1.06	−2.23 ± 0.25	21.98 ± 1.90	−4.02 ± 0.25
**DrugGPT M1**	−102.32 ± 6.25	−47.64 ± 5.66	−57.58 ± 3.59	−35.64 ± 1.75	−3.99 ± 0.51	50.59 ± 4.59	−3.00 ± 0.18
**DrugGPT M 2**	−99.92 ± 4.24	−30.62 ± 3.27	−60.03 ± 2.53	−31.55 ± 1.19	−2.23 ± 0.21	27.46 ± 1.76	−3.57 ± 0.15
**DrugGPT M 3**	−102.76 ± 5.41	−24.55 ± 2.59	−63.66 ± 3.13	−36.56 ± 1.92	−1.51 ± 0.15	27.48 ± 1.97	−3.42 ± 0.18
**Triazole-1**	−96.81 ± 5.71	−16.75 ± 3.40	−62.94 ± 3.44	−38.97 ± 2.53	−0.80 ± 0.33	26.85 ± 2.09	−2.93 ± 0.17
**Triazole-2**	−89.30 ± 4.26	−89.36 ± 4.73	−52.72 ± 3.36	−21.23 ± 1.04	−2.30 ± 0.44	81.98 ± 4.27	−3.31 ± 0.16

## Data Availability

The raw data supporting the conclusions of this article will be made available by the authors on request.

## Supplementary Materials

The following supporting information can be downloaded at: https://www.mdpi.com/article/10.3390/ph19081269/s1. Figure S1. Structure of LasR with the representation of the highly dynamic regions (HDR) during simulation. Figure S2. Hydrogen bond distance and angle distribution analysis of LasR–ligand complexes. The figure shows the H-bond distance distribution for (a) control (6MWL), (b) LasR– DrugGPT M1, (c) LasR– DrugGPT M2, (d) LasR– DrugGPT M3, (e) LasR–TD-1, and (f) LasR–TD-2 complexes. The panel with blue bars represents the H-bond distance distribution, while the green bars represent the angle distribution. Figure S3. Time-dependent analysis of the hydrogen bond occupancy in each LasR–ligand complex over 500 ns MD simulations. The hydrogen bond occupancy is shown for LasR-control (6MWL), LasR– DrugGPT M1, LasR– DrugGPT M2, LasR–DrugGPT M3, LasR–TD-1, and LasR–TD-2 complexes. The occupancy is shown as a heatmap, with a color-coded panel to indicate bonding occupancy.
