# Supplementary material for: Combining Triazole Scaffold Repurposing and Generative Transformer Architecture for Structure-Based Inhibitor Design Targeting the LasR Quorum Sensing Receptor of Pseudomonas aeruginosa
Source: Pharmaceuticals (Basel). 2026 Aug 11;19(8):1269. doi: 10.3390/ph19081269 (PMC13516403; doi:10.3390/ph19081269)
Supplement: Supplementary file 1 [file pharmaceuticals-19-01269-s001.zip › pharmaceuticals-4289621-supplementary.pdf]

Supplementary Materials

Article

Combining Triazole Scaffold Repurposing and Generative Transformer Architecture for Structure-Based Inhibitor Design Targeting the LasR Quorum Sensing Receptor of *Pseudomonas aeruginosa*

Abbas Khan [1], Muhammad Ammar Zahid [1], Anwar Mohammad [2], Asia Al-Jabiry [3], Raed M. Al-Zoubi [4,5,6], Mohanad Shkoor [3], Ameera Al-Jabiry [3]* and Abdelali Agouni [1]*

1. Department of Pharmaceutical Sciences, College of Pharmacy, QU Health, Qatar University, P.O. Box 2713, Doha, Qatar.
2. Precision Health Analysis Unit, Translational Research, Dasman Diabetes Institute, Dasman, Kuwait.
3. Department of Chemistry and Earth Sciences, College of Arts and Sciences, Qatar University, P.O. Box 2713, Doha, Qatar.
4. Surgical Research Section, Department of Surgery, Hamad Medical Corporation, Doha, Qatar
5. Department of Biomedical Sciences, College of Health Sciences, QU Health, Qatar University, P.O. Box 2713, Doha, Qatar.
6. Department of Chemistry, Jordan University of Science and Technology, P.O. Box 3030, Irbid, 22110, Jordan

***Corresponding** authors: **Prof.** Abdelali Agouni, Department of Pharmaceutical Sciences, College of Pharmacy, QU Health, Qatar University, P.O. Box 2713, Doha, Qatar. **Email:** aagouni@qu.edu.qa. **Dr.** Ameera Al-Jabri, Department of Chemistry and Earth Sciences, College of Arts and Science, Qatar University, P.O. Box 2713, Doha, Qatar; **Email:** a.aljabiry@qu.edu.qa

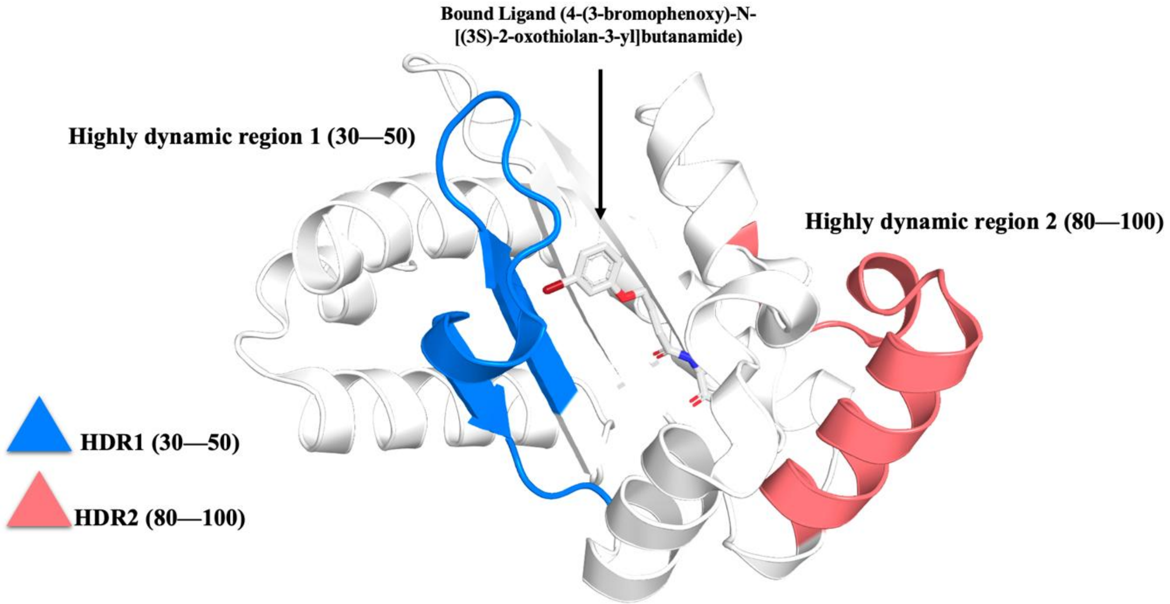

Figure S1: Structure of LasR with the representation of the highly dynamic regions (HDR) during simulation.

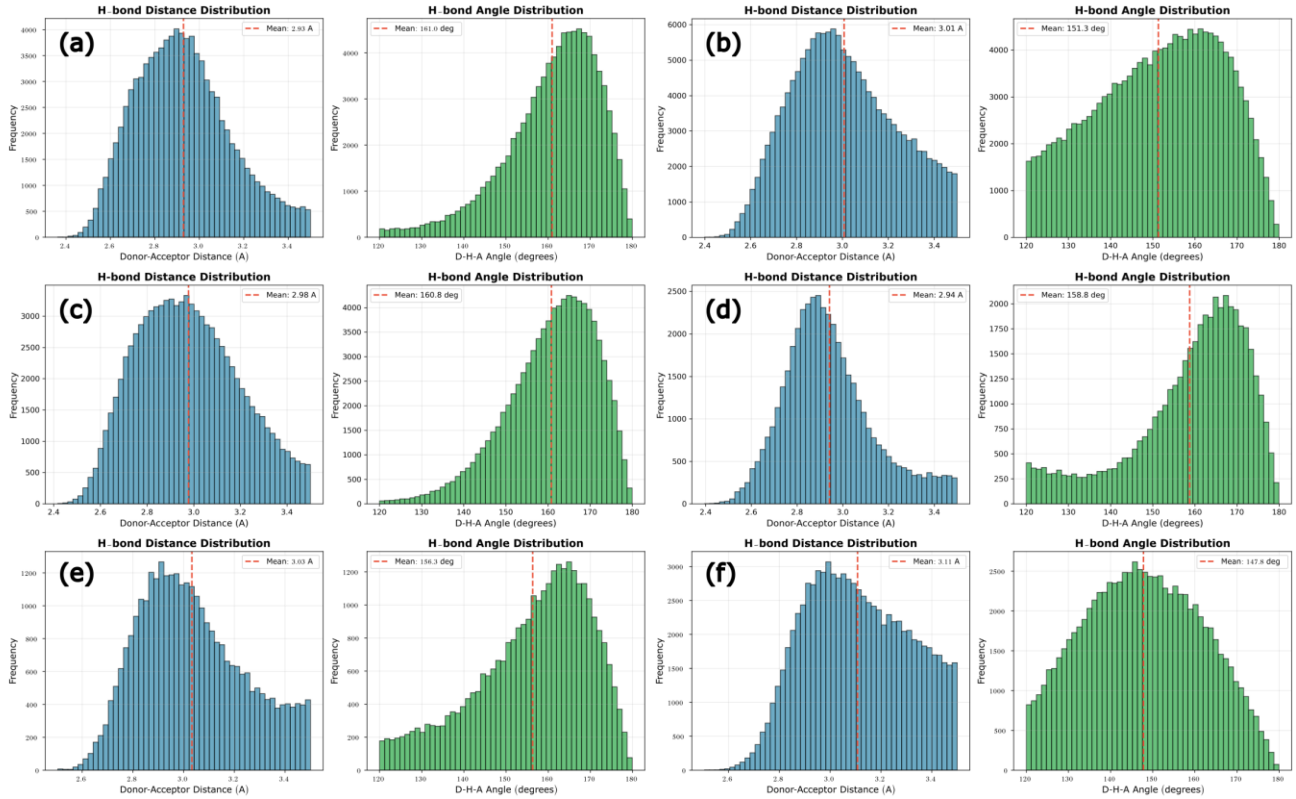

Figure S2. Hydrogen bond distance and angle distribution analysis of LasR–ligand complexes. The figure shows the H-bond distance distribution for **(a)** control (6MWL), **(b)** LasR– DrugGPT M1, **(c)** LasR– DrugGPT M2, **(d)** LasR–DrugGPT M3, **(e)** LasR–TD-1, and **(f)** LasR–TD-2 complexes. The panel with blue bars represents the H-bond distance distribution, while the green bars represent the angle distribution.

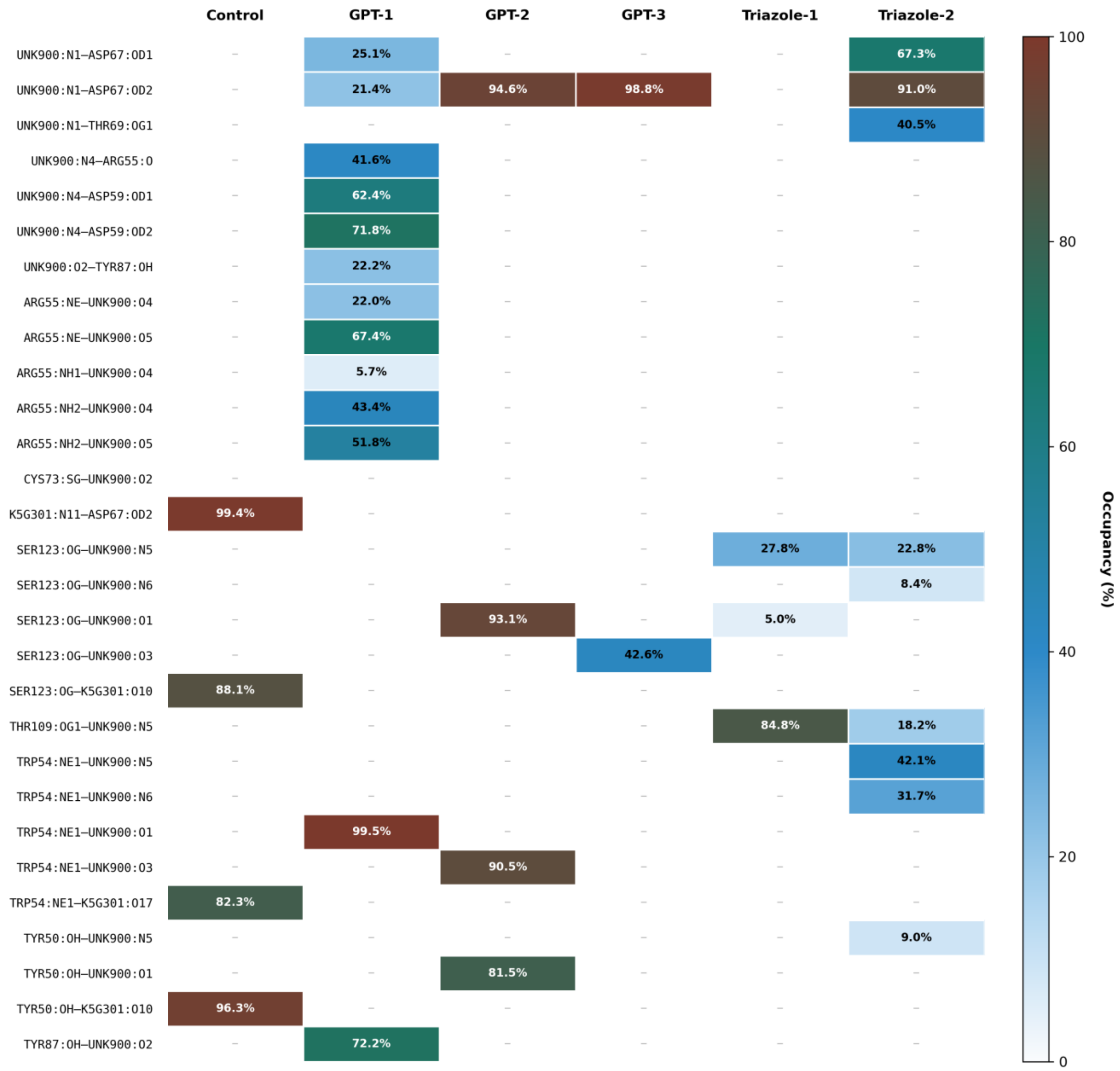

Figure S3. Time-dependent analysis of the hydrogen bond occupancy in each LasR–ligand complex over 500 ns MD simulations. The hydrogen bond occupancy is shown for LasR-control (6MWL), LasR– DrugGPT M1, LasR– DrugGPT M2, LasR–DrugGPT M3, LasR–TD-1, and LasR–TD-2 complexes. The occupancy is shown as a heatmap, with a color-coded panel to indicate bonding occupancy.
